# Cutting-Edge Sensor Technologies for Exosome Detection: Reviewing Role of Antibodies and Aptamers

**DOI:** 10.3390/bios15080511

**Published:** 2025-08-06

**Authors:** Sumedha Nitin Prabhu, Guozhen Liu

**Affiliations:** 1 Biomedical Engineering Programme, School of Medicine, The Chinese University of Hong Kong, Shenzhen 518172, China; liuguozhen@cuhk.edu.cn; 2 Guangdong Basic Research Center of Excellence for Aggregate Science, School of Science and Engineering, Shenzhen Institute of Aggregate Science and Technology, The Chinese University of Hong Kong, Shenzhen 518172, China

**Keywords:** exosomes, biomarker, aptamer, antibody, antigen

## Abstract

Exosomes are membranous vesicles that play a crucial role as intercellular messengers. Cells secrete exosomes, which can be found in a variety of bodily fluids such as amniotic fluid, semen, breast milk, tears, saliva, urine, blood, bile, ascites, and cerebrospinal fluid. Exosomes have a distinct bilipid protein structure and can be as small as 30–150 nm in diameter. They may transport and exchange multiple cellular messenger cargoes across cells and are used as a non-invasive biomarker for various illnesses. Due to their unique features, exosomes are recognized as the most effective biomarkers for cancer and other disease detection. We give a review of the most current applications of exosomes derived from various sources in the prognosis and diagnosis of multiple diseases. This review also briefly examines the significance of exosomes and their applications in biomedical research, including the use of aptamers and antibody–antigen functionalized biosensors.

## 1. Introduction

Exosomes are tiny membrane entities, possibly at the nanoscale. Exosomes were found in extracellular space during the late 1980s [1]. Exosomes, on the other hand, were traditionally thought to be intracellular vestigial products caused by cell damage or the consequences of cell homeostasis. Initially, they were viewed as waste entities with no significant impact on neighboring cells. These extracellular vesicles were recently shown to be functional vehicles capable of conveying a mixture of nucleic acids [2,3], proteins [4], and lipids [5]. Exosomes will carry these components to the cells they encounter. This mechanism, together with the recipient cells’ distal release functionality, may gradually reprogram the distant cells [6]. As a result, exosomes serve as a unique intercellular messenger, playing a crucial role in various cellular functions. These processes include the immune response [7], signal transduction [8], and antigen presentation [9].

**Figure 1:**
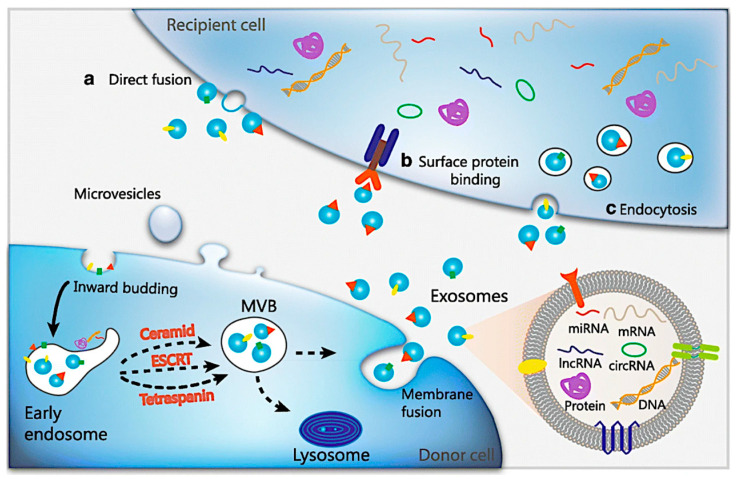
The biosynthesis and exosome contents [14]. The inward budding of the plasma membrane leads to the formation of early endosomes with membrane proteins incorporated. Then, the invagination of endosomes and the enclosing of selected cargos, including nucleic acids and proteins, results in the generation of multivesicular bodies (MVBs) through either ESCRT-dependent or ESCRT-independent mechanisms. Subsequently, these MVBs fuse with the plasma membrane and release exosomes into the extracellular space. Exosomes release these cargos (proteins, mRNAs, miRNAs, lncRNAs, circRNAs, and DNAs) to the recipient cells via mechanisms including (**a**) direct fusion, (**b**) binding with surface proteins, and (**c**) endocytosis.

Cells communicate with one another through cellular contacts, such as gap junctions and superficial protein-to-protein connections. Cells communicate with long-distance cells using secreted chemicals. These chemicals include compounds such as hormones and cytokines, which facilitate signal transmission [10]. Furthermore, chemical and electrical signals are used during interaction [11]. Exosomes, which transport cell-specific nucleic acids, proteins, and lipids, are now recognized as a distinct intercellular communication route. This notion was founded on the discovery that exosomes produced by parental cells can interact with target cells, altering their behavior and phenotypic characteristics [12]. Exosomal biological applications rely greatly on the efficient delivery of genetic components. This can be achieved via receptor–ligand interactions, membrane mixing, or endocytosis (Figure 1) [13,14]. Exosomes are ingested by the target cells’ internal endosomal limiting membrane once they have merged with them. When the exosome is ingested, it causes horizontal gene transfer. The genes are transferred from the parental cell’s content to the cytoplasm of the target cells. The bioactive chemicals found in exosomes primarily influence target cells via three mechanisms. It first acts by directly stimulating target cells with ligands linked to their surfaces. It also affects the transmission of active receptors to recipient cells. Alternatively, it alters the recipient cells’ genomes through epigenetic remodeling by transferring functional RNAs, proteins, and lipid molecules [15]. Exosome amplification enables parental cells to interact with specific adjacent or distant target cells.

Exosomes have gained prominence as a therapeutic platform in recent decades due to their unique ability to transport and distribute internal cell-specific biochemical information, as well as communicate with target cells [16,17]. Chemical transport is used by cells to interact with nearby cells, non-neighboring cells, and distant organs, which is frequently accomplished through the extracellular vesicle (EV) system. It carries organic molecules, including genomic DNA, RNA, messenger RNA, non-coding RNA, carbohydrates, bioactive lipids, microRNA, and cell-specific or membrane proteins [18,19]. Numerous studies show that exosomes enter cells and transport chemical messengers. Exosomes play a role in cell consolidation in various physiological and pathological conditions [20]. EVs are divided into three groups according to size and origin. They are classified into three sizes: exosomes (30–150 nm), microvesicles/ectosomes (100–1000 nm), and apoptotic bodies (50–5000 nm) [21].

Exosomes are generated through exocytosis, which involves the inward protrusion of the limited multivesicular body (MVB) membrane. The MVB is produced later on from an early endosome. Intraluminal vesicles (ILVs) arise inside big MVBs as a result of late endosomal membrane invagination [22]. Various proteins are introduced to the MVB membrane during the ILV development process. The water-soluble components of cell cytoplasm are also absorbed and accumulate inside the ILVs. As ILVs fuse internally with the mother cell’s plasma membrane, their internal exosomes are released into the extracellular area [23,24]. If unused, these components are transferred to lysosomes (Lyz) for intracellular degradation [25].

Exosomes are membranous vesicles that are discharged outside of the mother cell as a result of the cellular membrane’s outward budding during the late stage of endosomes [26]. Exosomes in physiological fluids have been shown in multiple studies to be useful indicators for disease prediction. Exosome molecular components are thought to play a role in the development and recovery of many diseases. Exosomes are produced spontaneously and actively by almost all eukaryotic cell types, including epithelial cells, macrophages, mast cells, neurons, tumor cells, oligodendrocytes, T cells, B cells, dendritic cells, platelets, mesenchymal cells, and mastocytes [2]. Exosomes can be found in amniotic fluid [27], semen [28], breast milk [29], tears, saliva [30], urine [31], blood, bile, ascites [32], and cerebrospinal fluid [33,34]. Exosomes perform critical biological functions. As a result, they can be found in many different bodily fluids. Exosomes contain proteins that facilitate the creation of vesicles and the transport of cellular signals [35]. Exosome content is very illness specific, particularly in cancer, Alzheimer’s disease, viral infections, and prion diseases. They exhibit the characteristics of their parent cells, indicating their cellular origin [36]. Exosomes possess several distinct properties, making them a potential biomarker. Exosomes play a crucial role in cell communication, immune responses, rejuvenation, and differentiation. Exosome signaling has also been connected to viral replication [37].

Exosomes have sparked researchers’ interest since they are predicted to facilitate intercellular communication and the transfer of macromolecules between cells. Second, throughout the present decade, exosomes have been connected to the spread of proteins [38], lipids [39], mRNA [40], miRNA [41], and DNA [42], ncRNA [42], noncoding RNA [42], as well known as the pathogenic and therapeutic cargo for many diseases [43]. Third, they have been proposed as drug vectors because they are composed of cell membranes rather than synthetic polymers, which are better tolerated by host cells [44].

This review study provides a brief discussion of exosomes and several functionalization strategies, including the structural features of aptamers [45] and antibodies [46]. This review also includes a wide range of cutting-edge compilations of research publications on functionalized sensors. It encompasses a wide range of disease-based exosome detection techniques that utilize aptamers or antibodies as intermediaries for target detection. This review encompassed a wide range of sensor technology types, including magnetic, SPR, SERS, lateral flow strip, luminescence, colorimetric, electrochemical, and fluorescent methods, applied to functionalized biosensors for the detection of various disease-specific exosomes using multiple methodologies. In the following paragraphs, we give insights, judgments, and pros and cons of the significance of the exemplary articles assessed in Section 3.

Although building exosome-based next-generation prognostic biosensors is extremely challenging, it is crucial to develop a rapid, early diagnostic, and prophylactic patient healthcare system for sustaining global wellness. Following a thorough review and extensive comparison of the available sensor types for aptamer detection, the authors reached a mutual conclusion. Scientists have confirmed a previously unknown potential for identifying chronic diseases using exosomes via magnetoelastic and giant magnetoresistance sensor-based detection techniques.

It also thoroughly discusses various instrumentation methods, including magnetic, surface plasmon resonance (SPR), surface-enhanced Raman scattering (SERS), lateral flow strip, luminescence, colorimetric, electrochemical, and fluorescent procedures, with a summary in Table 1. In conclusion, this review study discusses current details about various functionalization methods, exosome biosensing strengths, limitations, problems, opportunities for prospective applications, and future perspectives.

## 2. Techniques for the Separation of Exosomes as Well as Challenges in Exosome Separation from Body Fluids and Different Functionalization Methods

### 2.1. Traditional and Advanced Techniques for Exosome Isolation from Body Fluids

Isolating exosomes from various human body fluids, such as plasma, serum, urine, saliva, and cerebrospinal fluid (CSF), is a dynamic and expanding field of study that is critical to unlocking their diagnostic and therapeutic value. Traditional exosome separation protocols are largely based on differential ultracentrifugation, which is regarded as the gold standard due to its ability to process large sample volumes and produce relatively pure exosomal preparations by exploiting differences in particle size and density via sequential centrifugation at increasing speeds. The procedure requires pre-clearing biofluids with low- and medium-speed centrifugation to remove cells, debris, and bigger vesicles, followed by high-speed ultracentrifugation (≥100,000 g) to pellet the exosomal fraction. Density-gradient ultracentrifugation can separate exosomes based on buoyant densities (1.10–1.21 g/mL), resulting in highly purified fractions suitable for downstream omics and functional analyses [47,48]. However, ultracentrifugation-based approaches have limitations: they are labor intensive, time consuming, require expensive equipment, and may damage exosome integrity or result in low yields, particularly with viscous or restricted clinical samples. As a result, complementary and alternative size-based techniques such as size-exclusion chromatography (SEC), ultrafiltration, and field-flow fractionation (FFF) have been developed. SEC isolates vesicles based on hydrodynamic diameter as biofluids flow through porous polymeric matrices, efficiently separating exosomes (30–200 nm) from proteins and other contaminants while preserving vesicle function and morphology. Ultrafiltration uses molecular weight cutoff membranes and, through innovations such as tangential flow filtration (TFF), reduces membrane clogging and shear-induced vesicle damage. FFF, particularly asymmetric flow field-flow fractionation, enables quick, label-free subpopulation differentiation; however, implementation necessitates specialized equipment and experience [47,49]. Each approach has various trade-offs in yield, purity, scalability, and compatibility with clinical workflows, necessitating method selection based on sample type, downstream application, and clinical limitations.

### 2.2. Innovations and Challenges in Exosome Separation: Immunoaffinity and Microfluidic Approaches

To address the inherent constraints of classic isolation methods, such as inadequate specificity and co-isolation of contaminants, fresh technical developments have altered exosome separation from bodily fluids. Immunoaffinity-based methods, for example, use antibodies targeting exosome-specific surface proteins (such as CD63, CD9, and CD81) immobilized on solid supports, magnetic beads, or microfluidic platforms, allowing for the capture and enrichment of subpopulations of exosomes directly from complex matrices with unparalleled specificity and purity. This method is particularly useful for isolating disease- or tissue-specific exosome subsets, as demonstrated by the application of antibodies against tumor cell or neuron-specific markers in plasma or CSF. However, immunoaffinity capture can be expensive, rely on antibody quality, and result in reduced total vesicle recovery—factors that are closely analyzed in clinical translation [47,48]. Microfluidic technologies represent a quantum leap in exosome separation, combining size-based filtration, immunoaffinity capture, acoustic, and electrokinetic principles to create portable, automated, and high-throughput lab-on-chip systems. These microsystems enable exosome extraction from small sample quantities, rapid processing of many samples in parallel, and direct coupling to molecular assays for genomics or proteomics analyses. Acoustic microfluidic devices that distinguish exosomes via ultrasonically induced density and compressibility gradients, as well as field-flow fractionation chips that use viscoelastic flows for label-free isolation, are examples of notable advancements. The integration of surface plasmon resonance and electrochemical sensors enables real-time, label-free measurement and phenotyping of isolated exosomes down to the single-vesicle level [48,49]. Nonetheless, these developing approaches confront obstacles such as standardization, cross-platform repeatability, validation across diverse clinical samples, and scalability for routine diagnostic use. Critical pre-analytical parameters, such as sample collection, processing, and storage, have a significant impact on exosome yield and composition, necessitating strict protocol harmonization and quality control to fully realize the translational and therapeutic utility of exosome-based liquid biopsy [47,49].

### 2.3. Different Functionalization Methods

A sensor can be functionalized with aptamers or antibodies to detect the presence of exosomes in test samples. This section depicts the structures of aptamers and antibodies.

### 2.4. Aptamer

Aptamers are single-stranded synthesized RNA or DNA oligonucleotides. They attach to a variety of targets using three-dimensional interaction and structural complementarity, which yield precise identification and strong binding [50,51]. They bind to their targets in the same way that antibodies do, and they are known as “chemical antibodies” [52]. However, some aptamers have properties that make them excellent recognition components. Aptamers are quite inexpensive to produce. They can be chemically changed. Aptamers gain physical stability by chemical change. Aptamers denature reversibly when exposed to severe heat, although they remain functional across a wide pH range [53,54]. Aptamers are selected from combinatorial libraries of synthetic nucleic acids using in vitro techniques. It employs a method known as Systematic Evolution of Ligands by EXponential Enrichment (SELEX). A SELEX approach was employed to generate numerous high-affinity aptamers targeting various disease indicators. These high-affinity aptamers were used in sensor systems for disease detection [55].

### 2.5. Antibody

The antibody is a branched receptor, allowing several binding regions and complement activation [56]. The antibody structural model also included the idea of complementarity in antibody–antigen recognition. The antibody structural model is consistent with Fischer’s fit for the “lock and key” model of enzymes. In the succeeding decades, researchers have gained a thorough understanding of this molecule’s three-dimensional structure and function. Antibodies have a slanted Y-shaped structure with two arms, which are referred to as the Fab fragments [57,58,59]. The Fab fragments have equal antigen-binding sites and a stem. The stem is also called the Fc fragment, which is present at the tip regions of Fab fragments. The Fc fragment is linked with the Fab fragments using a flexible hinge. Every antibody molecule contains two identical chains. These chains are referred to as the heavy (H) chain and the light (L) chain [60,61,62,63]. Each chain has an N-terminal variable (V) domain, also known as VH and VL. The VH and VL are individually present in heavy and light chains. There are many classes: light chains (κ and λ) and heavy chains (α, γ, δ, ε, and μ) [64,65]. The ε and μ chains contain four constant (C) domains, whereas α, γ, and δ chains have three constant domains. The domain structure is comparable to the variable and constant domains, commonly referred to as the immunoglobulin domain structure. The immunoglobulin structure may be found in numerous variations in members of a large superfamily of antibodies. In antibodies, the functional unit is often a pair of domains from separate chains closely connected by noncovalent connection combinations. Their combinations can be denoted as VH:VL, CH1:CL, and CH3:CH3 [56]. The antibodies are high-affinity molecules, and they were employed in sensor systems for disease detection.

## 3. Advanced Sensing Methods for Exosome Detection Using Aptamers and Antibodies

Although building exosome-based next-generation prognostic biosensors is extremely challenging, it is crucial to develop a rapid, early diagnostic, and prophylactic patient healthcare system for sustaining global wellness. Following a thorough review and extensive comparison of the available sensor types for aptamer detection, the authors reached a mutual conclusion. The scientists confirm a previously unknown potential for identifying chronic diseases employing exosomes via magnetoelastic and giant magnetoresistance sensor-based detection techniques.

Exosome detection can be carried out utilizing a variety of approaches. The methods are as follows: magnetic, SPR, SERS, lateral flow strip, luminescence, colorimetric, electrochemical, and fluorescent.

**Table 1:** Sensor types for aptamers and antibodies.

Sensing Methods	Mechanism	Target	LOD	Reference	Strengths	**Possible Limitations**
Magnetic	Magnetic separation approach	Cell-exosome uptake and wound-healing using CD63 proteins	-	[66]	Lack of moving parts Capacity to detect their magnetic components through plastic, non-magnetic material, walls Small size Robustness in design Cost effective	Limitations while using sensors with ferrous materials Inability to function in high-temperature environments Detection range
	Labeled integrated magnetic analysis of glycans in extracellular vesicles	EV glycan role in natural biofluids against CD63 proteins	~10^4^ vesicles	[67]		
SPR	Dual AuNP-assisted amplification of the signal	MCF-7 breast cancer cells using CD63 proteins	5 × 10^3^ particles/µL	[68]	Non-destructive nature Quick and real-time accurate measurement of the targeted biomarker with great selectivity Repeatability High efficiency Detect both clear and colorful specimens Specific biomolecule detections from blood, urine, saliva, or plasma at low concentrations	The propagation length of the SPR wave is relatively short Sensor resolution is restricted by sensor system noise The operational range of intensity-measurement-based SPR sensors is inherently limited Commercial biosensors target a narrow segment of the (bio)chemical industry
	Polydopamine-functionalized gold nanoparticle (Au@PDA NP)–assisted signal amplification	human hepatic carcinoma (SMMC-7721) cell line offered SMMC-7721 exosomes in liquid biopsy and they were detected using CD63 proteins	5.6 × 10^5^ particles mL^−1^	[69]		
	The laser beam is split into two beams by an optical splitter	Non-small cell lung cancer diagnostics using CD63 proteins	2 × 10^10^ exosomes/mL	[70]		
	Intravesicular nanoplasmonic system platform used nanohole-based SPR for molecular detection	Transmembrane (CD63, EpCAM, EGFR) proteins, were measured in ovarian cancer cell lines	10^4^ EVs	[71]		
	Sandwich approach for the detection of clinically relevant exosomes	Isolate bulk exosomes and detect ≥10% HER2(+) exosomes in mixed samples; 14–35% HER2(+) exosomes in patient samples using CD9 and CD63 proteins	2070 exosomes/μL	[72]		
	Plasmonic biosensor for the analysis of EVs based on GC-SPR with wavelength interrogation	mesenchymal stem cell EVs using CD81 protein	670 aM	[73]		
SERS	AuNPs for targeting exosomes and a Raman reporter for signal readout source	Breast, colorectal, and prostate cancers detection using CD63 proteins	32, 73, 203 particles/µL	[74]	Capability to pique interest as an analytical method for chemical and biological applications Simple operation without extensive sample preparation Single-molecule sensitivity High throughput Point-of-care applications using widely accessible portable Raman spectroscopes LSP effects can provide electric and magnetic field enhancements that are many orders of magnitude greater than the incident field Repeatability Label-free detection	Slight variations in nanoparticles cause considerable changes in SERS intensity Scattering Raman light by a single molecule is time-consuming A Raman cross-section might be calculated to be nearly a million times smaller than a fluorescence cross-section
	Gold–silver bimetallic SERS-active nanotags nanotrepangs and a Raman reporter	Various exosome species for cancer	26, 72, 35 particles/μL	[75]		
	Sandwich-type immunocomplex	CD9 protein on the surfaces of exosomes using CD9 proteins	27 particles/µL	[76]		
	H-SERS substrate and a rapid enrichment strategy MEDP	LRG1-Exosomes and GPC1-Exosomes were selected to discriminate against pancreatic cancer using CD63 proteins	15 particles µL^−1^	[77]		
Lateral Flow Strip	Sandwich strip assay	CD63 protein antigen against different clinical samples	1.4 × 10^7^ particle/μL	[78]	Low-cost sensing Rapid results Portability Legible results by sight Simple operation Storage at room temperature Lengthy shelf life without electricity Various digital readers are available The readers can improve the accuracy of the result/recording, and it can be multiplexed	Samples containing particulates/viscous samples might cause blockage or inconsistent device operation Pretreatment of specific sample types may be required It may be less accurate (lower sensitivity/specificity) than lab-based tests After test completion, they must be properly disposed of while possibly harboring infectious material Although certain benchtop readers may accommodate several devices Normally, they must be read within a specific time limit since the effect will fade or overdevelop if left too long The sensor provides a “yes” or “no” response and is often not quantitative (unless for digitally read devices) Readers increase the difficulty and cost of assay production for customers The reproducibility of a batch might vary Cross-reactivity can be difficult, especially with multiplex devices
	Competitive strip assay	CD63 protein on exosome from human lung carcinoma cells	6.4 × 10^9^ particles/mL	[79]		
	Sandwich strip assay	Exosomes from fetal bovine serum using CD9 proteins	1.3 × 10^3^ particles/μL	[80]		
	Non-competitive strip assay	Malignant melanoma cell line using CD9, CD 63 and CD81 mixed proteins	8.54 × 10^5^ exosomes/mL	[81]		
Luminescence	Electrochemiluminescence emitters of Ru(dcbpy)_3_^2+^ along with sandwich format	Exosomes-related disease diagnosis using CD63 proteins	37 particle/μL	[82]	Identifying targets that might otherwise go undetected The sensor can mark a target only visible when exposed to UV light It can differentiate between fluorescent and other highly reflecting materials Specific and capable of distinguishing between closely similar targets Sensitive to modest quantities of target within a dense background matrix High affinity and the ability to sustain binding even after multiple washing procedures Stable enough to be used for an extended period	The lack of selectivity when investigating multicomponent samples
	UCNPs as a donor and TAMRA as an acceptor	Exosomes derived from breast cancer cells using CD63 proteins	80 particles/μL	[83]		
	Autonomous microlaser formation with liquid crystal microdroplets undertaking micellar solubilization in a surfactant	Extracellular biomarkers in circulating biological fluids using CD63 proteins	1.1 × 10^4^ particles/μL	[84]		
Colorimetric	Dual signal amplification, enzyme-induced silver deposition on Au NRs, and HCR process	Colon cancer LoVo cells and breast cancer exosomes from serum samples using CD63 proteins	1.6 × 10^2^ particles/µL (UV visible spectroscopy) and 9 × 10^3^ particles/µL (naked eyes)	[85]	Low cost Quick response time Ease of use More convenient procedure than volumetric or gravimetric processes Easily adjusted for automation It doesn’t need an expert Used to do quantitative analyses on colored substances A portable system	It cannot evaluate colorless molecules More sample is required for analysis A standard solution must be prepared It has a modest sensitivity The same colors from conflicting material may cause findings to be incorrect For more accurate analysis, the specific wavelength bandwidth may be necessary In uncontrolled situations, tampering with the matrix might lead to depraved outcomes
	HRP-mimicking DNAzyme, the hemin/G-quadruplex toward H_2_O_2_ reduction	Breast cancer exosomes using CD63 proteins	3.94 × 10^5^ particles/mL	[86]		
	Au-NPFe_2_O_3_NC functionalized with a generic tetraspanin antibody-modified, screen-printed electrode with PLAP	Placental cell-derived exosomes release using CD63 proteins	10^3^ exosomes/mL	[87]		
	PDA conjugate polymer with distinctive optical characteristics	Exosomes isolated from the human plasma using CD63 proteins	3 × 10^8^ vesicles/mL	[88]		
Electrochemical	Immobilization-free dual-aptamer identification sensing method and hyperbranched DNA superstructure signal amplification	Isolation and quantification of tumor exosomes using CD63 proteins	3.0 × 10^4^ particles mL^−1^	[89]	Ability to be tailored particularly for a given analyte and their range in the sample space The analyte concentration determines the degree of selectivity Straight performance Low power consumption Linear output High resolution Remarkable repeatability Precision Once calibrated to a known analyte concentration, the sensor will read a repeated target analyte properly Other cross-interferences do not affect the output Practical method, presence of other analytes in the environment does not affect the sensor’s life Most alternative analyte detection methods, including infrared and photoionization detectors, are less costly	A constant temperature range is required Short or restricted shelf life Shelf life of six months to one year The cross-affectability of distinct analytes The life span is reduced because of increased exposure to the target analyte
	SMRT biosensor comprised two split-a and split-b fragments	Tumor exosomes from SMMC-7721 (human hepatocellular carcinoma cell line)	1.5 × 10^6^ particles/mL	[90]		
	Sandwich-type reduction of HRP-oxidized TMB electrochemical immunoassay	Distinguishes between exosomes and other EVs using CD9 proteins	200 particles/µL	[91]		
	An electrochemical paper-based analytical instrument having electrode-bound antibodies	Exosomes captured with ovarian cancer-specific CA125 antibodies using CD9 proteins	9.3 × 10^7^ exosomes/mL	[92]		
Fluorescent	TdT mediated forming ultra-long poly T as the activator	NPC-derived exosomes using CD63 proteins	100 particles mL^−1^	[93]	Portability High sensitivity Selectivity Fast response Ease of use It is a more appealing alternative for field applications when only a limited number of biochemical samples are available	Sensor’s reliance on probes constrains them If unlabeled, it is incapable of autofluorescence Unexpected or novel structures cannot be noticed without prior identification and proper probe design or selection The use of many probes at the same time, or the presence of autofluorescent biomolecules, may cause interactions that reduce the efficiency of each probe, resulting in a worse signal-to-noise ratio Many chemicals occurring naturally within the target and other fluorophores can quench a fluorescence signal
	MIP and aptamer/GO-based FRET system-based optical sensing in a sandwich mode	Lyz proteins and exosomes using CD63 proteins	2.43 × 10^6^ particles/mL	[94]		
	ARGET ATRP polymerization	The early stage of lung cancer using exosomes using CD63 proteins	11,610 exosomes/mL	[95]		
	GNP–DNA–FAM signal amplification technology	Leukemia cell-derived exosomes using CD63 proteins	1 × 10^2^ particles/L	[96]		

### 3.1. Magnetic Biosensors

The principle of immunomagnetic isolation procedures is to collect exosomes by identifying the unique signature receptors on their surface utilizing magnetic beads (MB) coated with anti-marker antibodies. The advantages of this procedure include a small sample amount, great accuracy, and no chemical contamination.

The strengths of magnetic sensors can be listed as the lack of moving parts, the sensors’ capacity to detect magnetic components through plastic, non-magnetic materials, walls, their small size, robust design, and cost-effective sensing technology.

The magnetic sensors also have a few minor possible limitations. It includes limitations while using sensors with ferrous materials, the inability to function in high-temperature environments, and their detection-related range.

**Figure 2:**
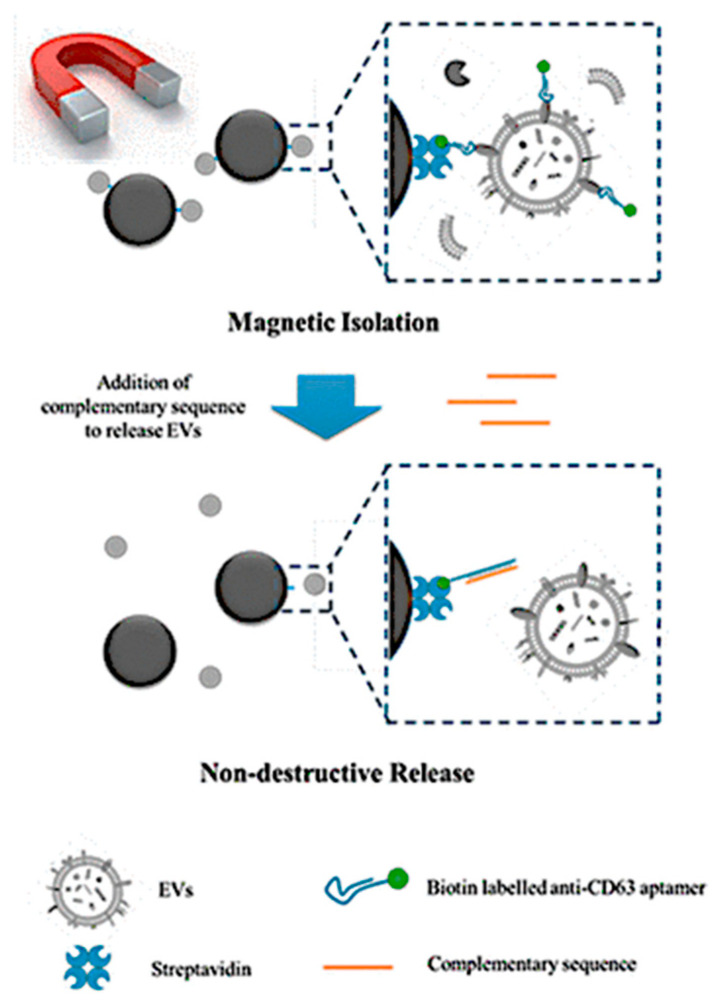
Schematic diagram of DNA AMI process for EV isolation. EVs from either cell culture medium or blood plasma were first marked with biotin-labeled CD63 aptamer and then separated using streptavidin-modified magnetic beads. After magnetic isolation, the complementary sequences, which can hybridize with the CD63 aptamer to break the aptamer secondary structure, were added for nondestructive release of the EVs from magnetic beads [66].

Zhang et al. developed a DNA aptamer-based approach that enabled the rapid capture and non-destructive release of EVs within 90 min while maintaining an isolation efficiency comparable to ultracentrifugation (approximately 78%). Furthermore, Zhang et al. used a DNA structure-switch technique to release the exosomes, and the separated EVs retained their high bioactivity in cell-uptake and wound-healing studies. Zhang et al. were able to separate EVs from clinical samples using magnetic technology. They discovered that the number of tumor biomarker MUC1-positive EVs in breast cancer patient plasma samples was much greater than in healthy donors. This magnetic separation approach, based on DNA aptamers, can be utilized for EV bio-function studies and EV-based point-of-care clinical testing (Figure 2) [66].

**Figure 3:**
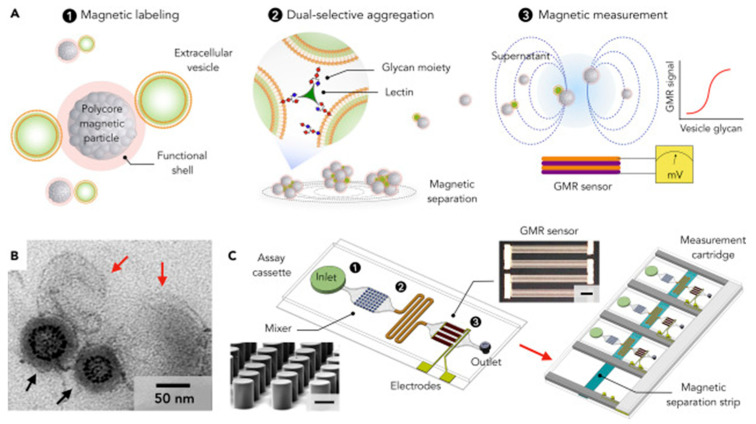
Integrated magnetic analysis of EV glycans [67]. (**A**) Schematic of the iMAGE analysis. The technology comprises three functional steps. EVs are first magnetically labeled with PMPs, which are size-matched and coated with a functional shell to improve binding with EVs. In the presence of specific glycan moieties on the vesicles, the addition of targeting lectins causes multivalent binding and aggregation of the EVs and their associated PMPs. This aggregation is dual-selective for both EV biophysical characteristics and specific glycans. As more EV glycan-specific aggregates form, they are sedimented by an external magnetic gradient and depleted from the solution. The remaining PMPs in the supernatant are measured through a real-time giant magnetoresistance (GMR) sensor and can be directly correlated to EV glycan profiles. (**B**) Transmission electron micrograph of EVs bound to PMPs. EVs derived from human kidney cancer cells (A498) were incubated with PMPs functionalized with polydopamine (PDA) to enable direct binding. EVs and PMPs are indicated by the red and black arrows, respectively. (**C**) Integrated magnetic analysis of EV glycans.

Wang et al. created a specialized analytical tool for directly profiling extracellular vesicle glycans in natural biofluids. The platform was labeled as integrated magnetic analysis of glycans in extracellular vesicles (iMAGE). It employs rationally designed polycore magnetic nanoparticles to convert extracellular vesicle-bound glycans into magnetic signals, but not free-floating glycoproteins; the resulting signals are easily quantifiable using a built-in magnetoresistance sensor. As a result, the iMAGE assay was quick (30 min), sensitive (10^4^ EVs), and wash-free. The method showed illness fingerprints against a complicated biological backdrop by multiplexing measurements of extracellular vesicle glycans in original clinical materials. It also distinguished patient prognosis. The measured iMAGE response correlated with extracellular vesicle counts and established an LOD of ~10^4^ vesicles (Figure 3) [67].

### 3.2. Surface Plasmon Resonance (SPR) Biosensors

SPR is an optical and biological technique for sensing that can detect biomolecular interactions on metal surfaces in real time. This approach does not harm biomolecules and does not require the use of any markers.

The strengths of SPR sensors can be listed as their non-destructive nature, the ability to measure the targeted biomarker quickly and in real-time with great selectivity, and repeatability. SPR is recognized for its high efficiency, accuracy, and real-time measurement capabilities. It can detect both clear and colorful specimens. Even at low concentrations, SPR-based sensors may efficiently confirm the presence of specific biomolecules in biological fluids such as blood, urine, saliva, or plasma.

The SPR sensors also have some possible minor limitations. It includes limitations, with the propagation length of the SPR wave being relatively short. The sensor resolution is restricted by sensor system noise. The operational range of intensity-measurement-based SPR sensors is inherently limited. Commercial biosensors target a narrow segment of the (bio)chemical industry.

**Figure 4:**
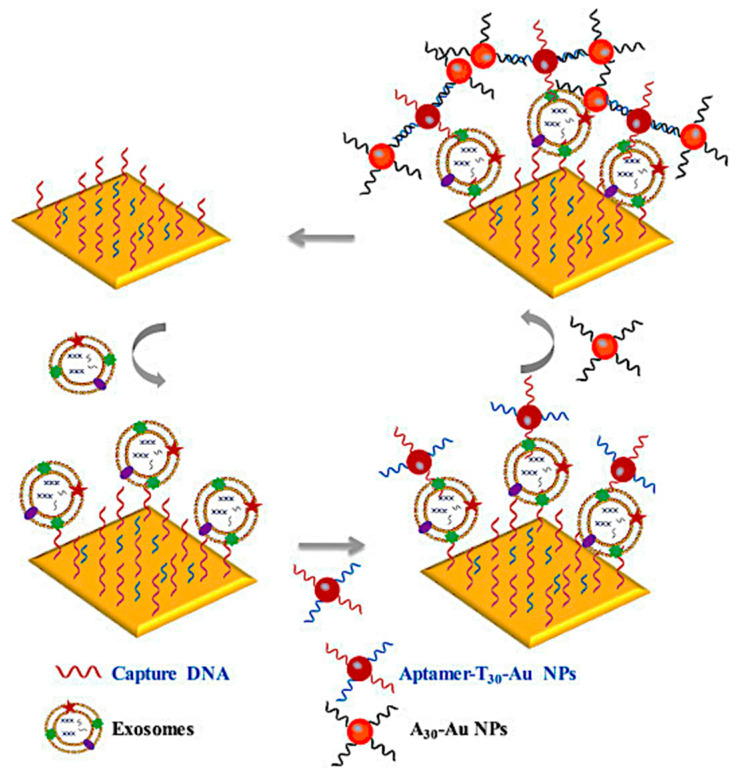
Dual AuNP-assisted signal amplification for determination of exosomes [68].

Wang et al. demonstrated the use of a sensitive aptamer sensor for exosome detection via SPR with dual gold nanoparticle (AuNP)-assisted signal amplification. Controlled hybridization attachment of AuNPs is caused by electronic coupling between the Au film and AuNPs. The coupling effects in plasmonic nanostructures were used to create dual nanoparticle amplification. Nonspecific adsorption of AuNPs onto the SPR chip surface was prevented by blocking the Au film surface with 11-mercapto-1-undecanol, and the SPR sensor was regenerated. This approach proved extremely sensitive, with a 5 × 10^3^ exosomes/mL LOD. The value of LOD represented a 10^4^-fold improvement over commercial ELISA (Figure 4) [68].

Liao et al. introduced a novel SPR strategy for identifying exosomes using aptamer recognition and polydopamine-functionalized gold nanoparticle (Au@PDA NP) signal amplification. Exosomes from hepatic cancer SMMC-7721 were used as a model target. The aptamer ZY-sls, which is complementary to DNA tetrahedron probes (DTPs), captures SMMC-7721 exosomes. CD63 aptamer-linked Au@PDA NPs subsequently identify and amplify the signal. DTPs were modified with an Au film to prevent surface deposition during HAuCl_4_ introduction. PDA-coated AuNPs were then utilized to reduce HAuCl_4_ in situ without the need for a reductant. It results in a stronger SPR signal. The assay distinguishes SMMC-7721 exosomes from other exosomes, including HepG2, Bel-7404, L02, MCF-7, and SW480. Exosomes from SMMC-7721 can be found at concentrations as low as 5.6 × 10^5^ particles/mL^−1^. The approach successfully detected SMMC-7721 exosomes in 50% of human serum without pretreatment [69].

**Figure 5:**
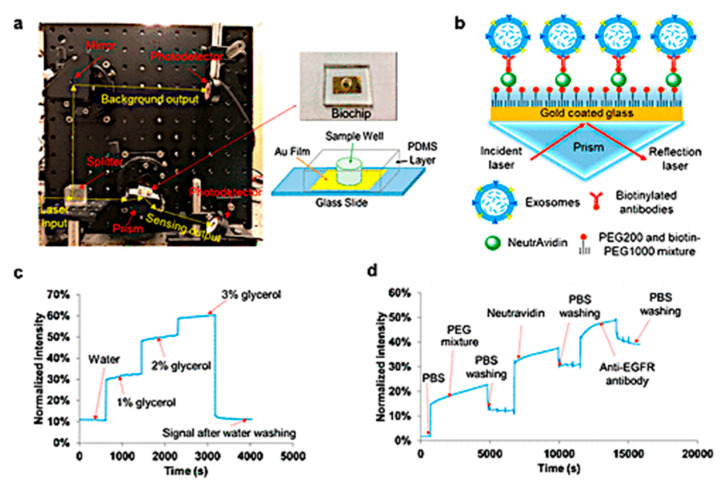
Schematic representation of a sensitive detection of exosomal proteins via a compact surface plasmon resonance biosensor for cancer diagnosis. (**a**) Setup of compact SPR biosensor (**left**) and the photo and schematic diagram of the biochip (**right**). (**b**) Sensing mechanism of compact SPR biosensor. (**c**) Calibration of sensing performance of compact SPR biosensor with 1%, 2%, and 3% glycerol solutions. (**d**) Real-time SPR response upon the addition of PBS, PEG mixture, Neutravidin, and anti-EGFR antibodies during the biochip surface modification [70].

Liu et al. created an intensity-harmonized, small SPR biosensor (25 cm × 10 cm × 25 cm) that employs a typical SPR sensing method and does not need the production of nanostructures. After calibration with glycerol, the small SPR biosensor demonstrated a sensitivity of 9.258 × 10^3^%/refractive index unit (RIU) and a resolution of 8.311 × 10^−6^ RIU. Liu et al. used exosomal epidermal growth factor receptor (EGFR) and programmed death-ligand 1 (PD-L1) as biomarkers to show the practicality of the compact SPR biosensor in lung cancer diagnostics. It found more exosomal EGFR in A549 non-small cell lung cancer (NSCLC) cells than in BEAS-2B normal cells. The compact SPR biosensor revealed identical amounts of exosomal EGFR in NSCLC patients and normal controls and greater expression of exosomal PD-L1 in NSCLC patients than normal controls in human serum samples. The small SPR biosensor outperformed ELISA in terms of detection sensitivity and sensing accuracy. The compact SPR biosensor successfully detected exosomal EGFR in A549 exosomes at the concentration LOD as 2 × 10^10^ exosomes/mL with a signal-to-noise ratio of 27 and a coefficient of variation of 16.6% (Figure 5) [70].

EVs, including exosomes, are nanoscale membrane particles released by cells that contain biological proteins that may help in cancer diagnosis and treatment. The majority of analyses have focused on surface proteins, whereas analyzing intracellular proteins has proven more difficult. Park et al. described an EV screening test for both intravesicular and transmembrane proteins that employed a nanoplasmonic sensor. This platform, known as the intravesicular nanoplasmonic system (iNPS), detects molecules using nanohole-based SPR technology. The authors developed a single test procedure to detect both intravesicular and transmembrane proteins, and then built plasmonic substrates to improve detection sensitivity. The resulting iNPS enables sensitive (0.5 μL sample per marker) and high-throughput (a 10 × 10 array) detection of EV proteins. When used to track EVs from drug-treated cancer cells, the iNPS assay revealed drug-dependent EV protein signatures. The authors believe that iNPS could be an effective technique for thorough molecular screening of EVs [71].

Sina et al. found that tumor-derived exosomes are clinically relevant due to their distinct genetic and protein makeup, which is similar to the parent tumor. Isolating and identifying therapeutically relevant exosomes from biological samples can enhance our understanding of their role as cancer biomarkers. The authors provided a straightforward method for determining the quantity of clinically relevant exosomes in patient serum. The fraction of clinically important exosomes can provide insight into disease stage and allow for non-invasive monitoring of tumor-receptor expression levels across individuals. The researchers used an SPR platform to measure the proportion of clinically relevant exosomes in a two-step process. This involved isolating bulk exosomes using biomarkers (e.g., CD9, CD63) and then detecting clinically relevant exosomes using tumor-specific markers e.g., human epidermal growth factor receptor 2 (HER2). The authors isolated bulk exosomes and detected up to 10% HER2(+) exosomes from samples containing specific proportions of HER2(+) BT474 and HER2(−) MDA-MB-231 cell-derived exosomes. They successfully isolated exosomes from a small cohort of breast cancer patient samples and found that 14–35% of the population expresses HER2 [72].

Reiner et al. used a GC-SPR biosensor to analyze tiny lipid EVs with sensitivity. To analyze tiny levels of EVs in complicated liquid samples, magnetic nanoparticles are used to concentrate the target analyte on the sensor surface. The affinity binding is then investigated utilizing wavelength interrogation of SPR. The GC-SPR technique effectively pulls EVs to the sensor surface utilizing magnetic nanoparticles and an external magnetic field gradient provided through the device. This method overcomes slow diffusion-limited mass transfer and significantly improves sensor response. A SPR sensor chip modified with antibodies against the surface marker CD81 and magnetic nanoparticles binding the vesicles via annexin V and cholera toxin B chain detects various EV populations secreted by mesenchymal stem cells [73].

### 3.3. Surface-Enhanced Raman Scattering (SERS) Biosensors

SERS possesses distinct properties that enable the detection of various exosomes, characterized by their restricted spectral bandwidth.

The strengths of SERS sensors can be listed as a capability to pique interest as an analytical method for chemical sensing and biological applications. SERS sensors have several benefits for analysis, including the capability to pique interest as an analytical method for chemical and biological applications, simple operation without extensive sample preparation, and single-molecule sensitivity. The SERS technique also offers high throughput, with point-of-care applications using widely accessible, portable Raman spectroscopes, along with localized surface plasmon (LSP) effects that can provide electric and magnetic field enhancements many orders of magnitude greater than the incident field. The SERS also offers repeatability and label-free detection.

The SERS sensors also have some possible minor limitations. Slight variations in nanoparticles cause considerable changes in SERS intensity in the case of SERS sensors. Although Raman Spectroscopy may discover a molecule’s unique chemical signature, scattering Raman light by a single molecule is time-consuming. In the event of the most advantageous resonant Raman scattering settings, a Raman cross-section might be calculated to be nearly a million times smaller than a fluorescence cross-section.

**Figure 6:**
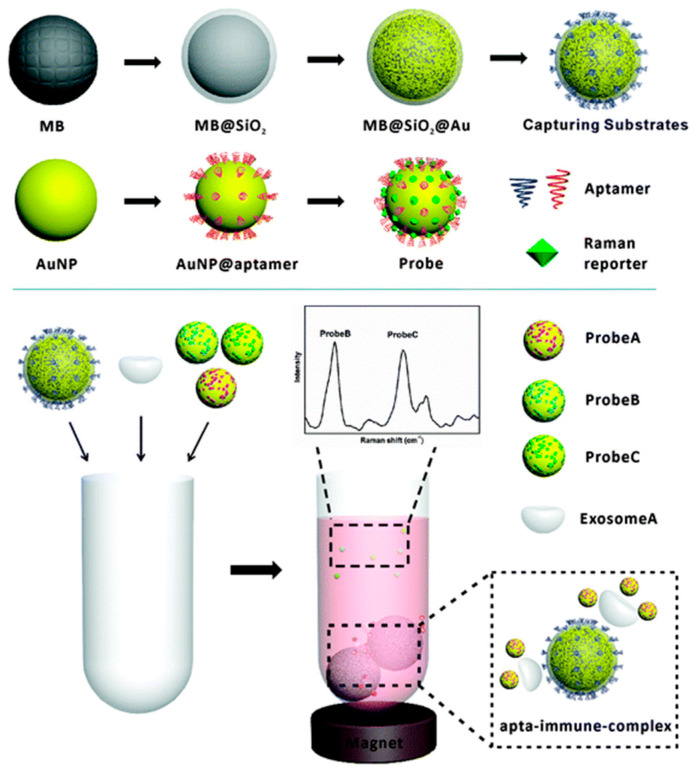
The principle of the SERS-based detection method [74].

Wang et al. created a SERS aptamer sensor that can detect numerous exosomes at the same time by combining various aptamer probes and MB capture substrates. The CD63 aptamer was coated with gold shell magnetic nanobead substrates to collect common exosomes. SERS identification was based on specialized aptamers modified AuNPs for targeting exosomes and a Raman reporter as a readout signal source, resulting in a sandwich of MB–exosome–AuNPs. The SERS signal in the supernatant dropped after magnetic separation. In this assay, three types of SERS probes with respective SERS spectra-dominant bands were used for multiple detections of three types of exosomes from breast, colorectal, and prostate cancers, with LOD values of 32, 73, and 203 particles/μL, respectively (Figure 6) [74].

**Figure 7:**
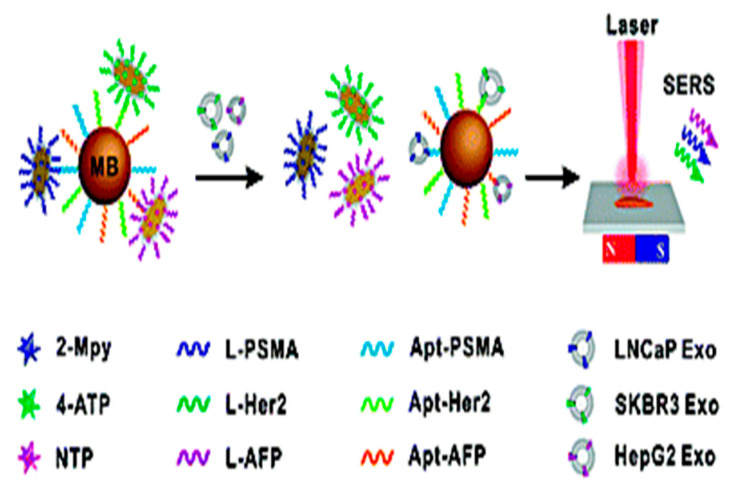
Schematic illustration of the fabrication of the SERS sensor for multiplex exosome detection. Three kinds of SERS detection probes were prepared via the functionalization of linker DNAs on 2-Mpy, 4-ATP, and NTP-encoded GSSNTs. Multiplexed capture probes were constructed by co-modification of three types of aptamer DNAs. Each kind of GSSNT was captured by the corresponding aptamer DNA anchored on MBs, followed by the recognition between aptamers and target exosomes, leading to the released GSSNTs and attenuated SERS signals [75].

Ning et al. created a sensitive and dependable SERS sensor. The SERS detection probes were formed of bimetallic SERS-active nanotags, gold–silver–silver core–shell shell nanotrepangs (GSSNTs), which were composed of rough gold surface nanotrepang (GNT) cores and bilayer silver shells and adorned with linker DNAs complementary to the aptamer-targeting exosomes. Three types of SERS detection probes were created using various Raman reporter molecules and linker DNAs. Certain aptamers of the target exosomes were modified on MBs to generate the capture probes. SERS detection probes were connected to MBs via specific DNA hybridization in the absence of target exosomes, allowing for the use of aptamer-based SERS sensors. The aptamer selectively detected and trapped the exosomes in the presence of target exosomes, and GSSNTs were then released into the supernatant. As a result, diminished SERS signals on the MBs were found, confirming the presence of target exosomes. SERS technologies enable the differentiation of diverse exosome species in a single step, potentially acting as a tool for cancer diagnostics (Figure 7) [75].

**Figure 8:**
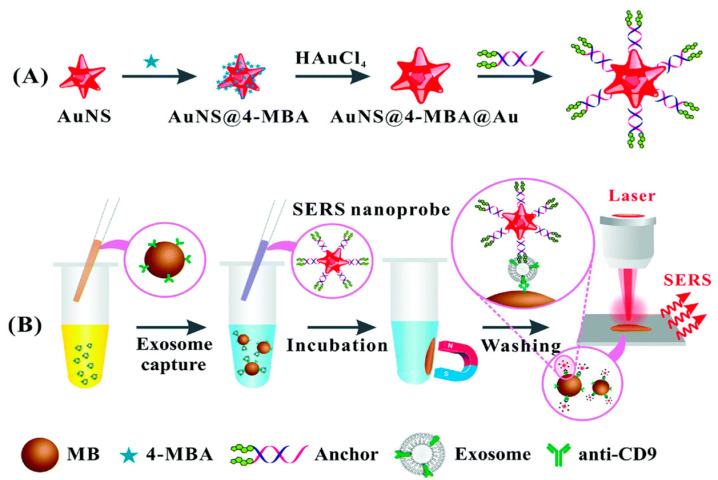
Sequential SERS-based assay process for the detection of exosomes. (**A**) Fabrication of SERS nanoprobes (AuNS@4-MBA@Au-anchor). (**B**) SERS sensing strategy for exosome detection [76].

Tian et al. presented a simple and highly sensitive SERS-based technique for selective extraction and measurement of exosomes that combines immunoaffinity, SERS nanoprobes, and portable Raman instruments. To create SERS nanoprobes, the surfaces of gold nanostar@4-mercaptobenzoic acid@nanoshell structures (AuNS@4-MBA@Au) are modified with bivalent cholesterol (B-Chol)-labeled DNA anchor. Exosomes are caught selectively by immunomagnetic beads, and SERS nanoprobes are subsequently fixed on the surface of exosomes via hydrophobic interactions between cholesterol and lipid membranes, generating a sandwich-type immunocomplex. Magnetic capture of the immunocomplex results in increased SERS signals. The sandwich-type immunocomplex cannot form in the absence of exosomes, resulting in low SERS signals. The degree of immunocomplex formation and the associated SERS signals are strongly connected with exosome concentration throughout a broad linear range of 40 to 4 × 10^7^ particles/µL, with 27 particles/µL LOD. As a result, a sensitive and easy technique for detecting exosomes was effectively developed (Figure 8) [76].

**Figure 9:**
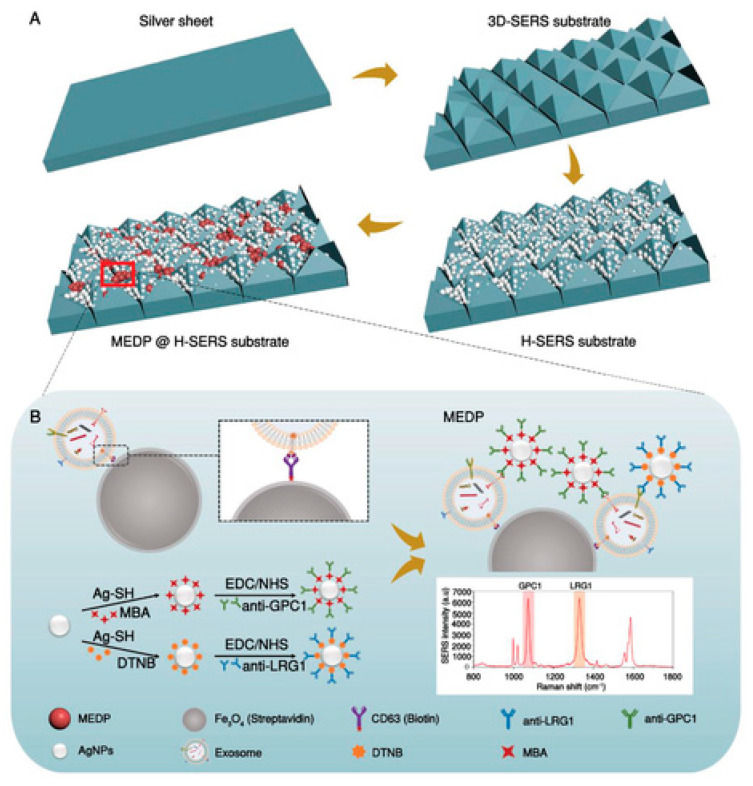
A highly sensitive exosome detection for early diagnosis of pancreatic cancer using immunoassay based on hierarchical surface-enhanced Raman scattering substrate. (**A**) The synthesis process of the H-SERS substrate. (**B**) Illustration of the construction of exosome capture system, the fabrication of SERS detection probes, and the SERS detection of exosomes [77].

Li et al. first constructed a very sensitive recognition system for directly quantitating specific exosomes in real samples in this study. They performed testing using a hierarchical-SERS substrate (H-SERS substrate) and a fast improvement strategy of magnetic beads @ exosomes @ SERS detection probe (MEDP). The detection system (MEDP @ H-SERS substrate) gave 3.5 times more SERS intensity than the MEDP sandwich immunocomplex alone. Additionally, by exosome proteomics and database screening, LRG1-positive exosomes (LRG1-Exosomes) and GPC1-positive exosomes (GPC1-Exosomes) were selected to discriminate pancreatic cancer. With the MEDP @ H-SERS substrate, the lowest LOD was determined to be 15 particles µL^−1^. Importantly, the detection in clinical samples demonstrates that the novel combination of LRG1-Exosomes and GPC1-Exosomes might increase the diagnostic efficiency of pancreatic cancer, with an area under the operating characteristic curve (AUC) of 0.95. Even in early-stage pancreatic cancer, diagnostic accuracy was good (AUC = 0.95). The findings suggest that the MEDP @ H-SERS substrate has considerable promise for the early detection of pancreatic cancer (Figure 9) [77].

### 3.4. Lateral Flow Strip Biosensors

The lateral flow strip aptamer sensor is typically developed on paper-based electronics with a simplified procedure. The outcome is immediately visible via color or signal change. The lateral flow strip is classified into three forms created on various contacts on the test line: sandwich strip, competitive strip, and non-competitive strip.

The strengths of lateral flow strip sensors are low-cost sensing, rapid results, portability, legible results by sight, and simple operation. They can normally be stored at room temperature, making them ideal for point-of-care and home testing, as well as testing in distant locations where refrigeration may be problematic or unreliable. Most sensors have a lengthy shelf life and do not require any electricity. Various digital readers are available to suit different scenarios, including those requiring small sample quantities. Readers can improve the accuracy of result interpretation and recording, and they can be multiplexed.

The lateral flow strip sensors also have some minor possibility of limitations. Samples containing particulates or viscous materials may cause blockage or inconsistent device operation. Pretreatment of specific sample types may be required. It may be less accurate (with lower sensitivity and specificity) than the lab-based tests. After test completion, they must be properly disposed of while possibly harboring infectious material. In lateral flow strip sensors, certain benchtop readers may accommodate several devices. Normally, they must be read within a specific time limit since the effect will fade or overdevelop if left too long. The sensor provides a “yes” or “no” response and is often not quantitative (unless for digitally read devices). Readers increase the difficulty and cost of assay production for customers. The reproducibility of a lateral flow strip sensor batch might vary. Cross-reactivity can be challenging, especially with multiplex devices.

**Figure 10:**
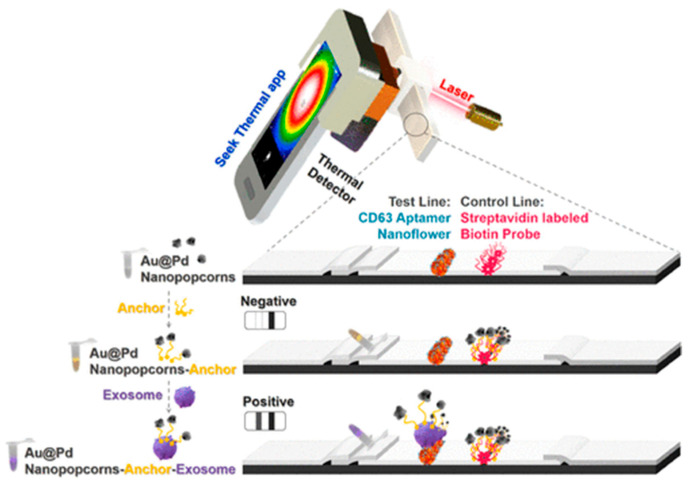
Schematic illustration of the strategy of integrating an ANAN-LFS with a smartphone-based thermal reader [78].

Cheng et al. developed a sandwich strip based on thermal signals for the identification of exosomes. In the sensor development, the Au@Pd nanopopcorn anchor was used. It was coupled with exosomes to generate the Au@Pd–exosome complex. The test line was enhanced with CD63 aptamer nanoflowers, which enabled the complex to be captured, resulting in a black band. The black band is owing to the buildup of nanopopcorn and a thermal signal through laser stimulation. The unbound exosome nanopopcorn would interface with the biotin-labeled probe at the control line. It resulted in the appearance of an additional black band. Cheng et al. also developed a thermal signal scanner for a portable smartphone with an LOD of 1.4 × 10^7^ particles/μL in view (Figure 10) [78].

**Figure 11:**
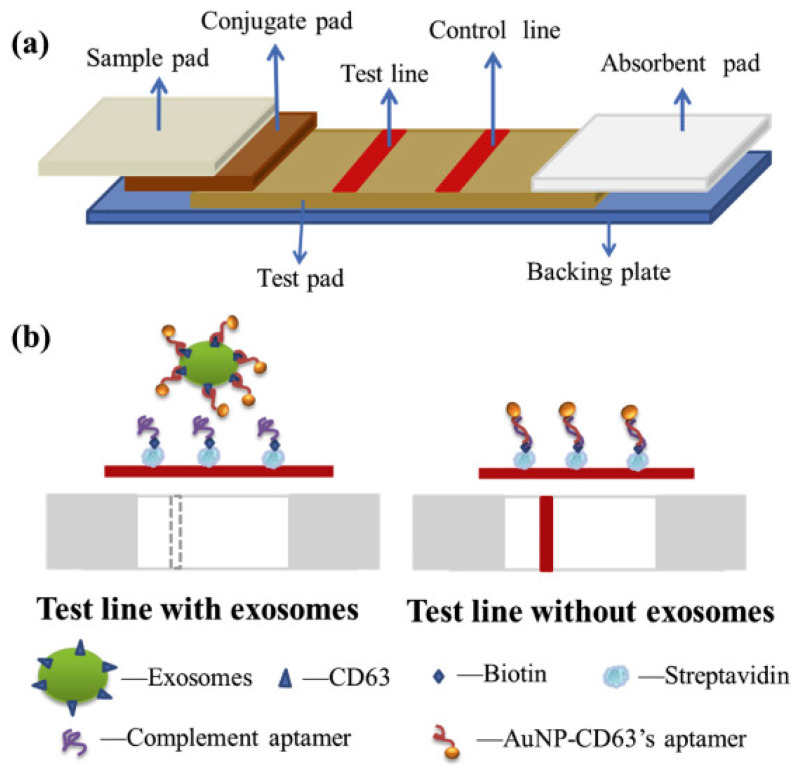
Configuration and principle of LFAA test strips. (**a**) Typical lateral flow test strips configuration. (**b**) Schematic diagram of the competitive LFAA test strips for exosome identification [79].

Yu et al. suggested a sensor based on a competitive strip for exosomal recognition to streamline the experimental process. Yu et al. purposefully eliminated the control line. Therefore, their test line showed the results immediately. CD63 aptamer attached-nanogold crossbred with its opposite sequence inserted at the test line without exosomes. This step resulted in turning the line red. Aptamers initially bound to exosomes in the presence of exosomes, and the unbound aptamer was connected to the test line, resulting in a pale coloration. As a result, the quantity of exosomes was inversely related to color. The lateral flow strip exhibited qualities such as ease of batch manufacturing, long-term stability, minimal sample requirements, and cost effectiveness, making it more suitable for clinical use. The LOD was 6.4 × 10^9^ particles/mL (Figure 11) [79].

**Figure 12:**
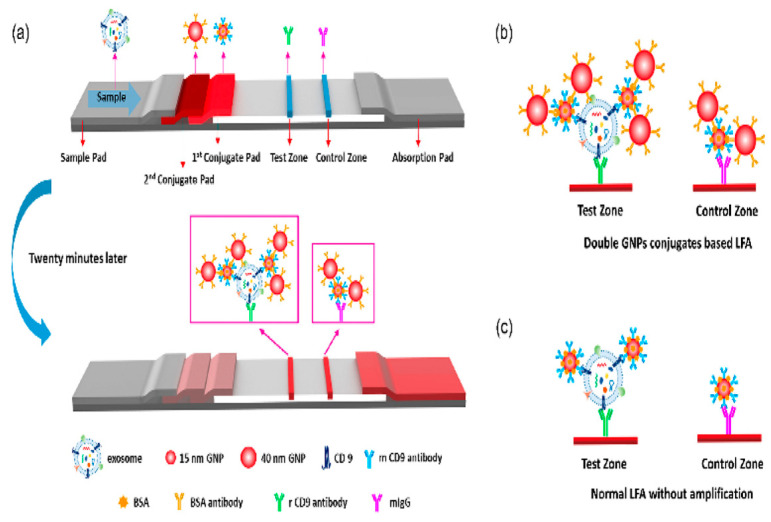
Enhanced lateral flow assay with double conjugates for the detection of exosomes. (**a**) Schematic illustration of the configuration and measurement principle of the D-LFAs and the result in the presence of exosomes. (**b**) The amplified structure on the test zone and control zone of the D-LFAs in the presence of exosomes. (**c**) The structure on the test zone and control zone of the normal LFAs without amplification in the presence of exosomes (color online) [80].

Wu et al. developed a lateral flow assay (D-LFA) based on double gold nanoparticle (GNP) conjugates for the rapid and sensitive detection and molecular characterization of exosomes. Signal amplification may be accomplished using only these two GNP conjugates and no extra operations. The antibody on the first GNP conjugate recognized exosomes on the test zone and formed a sandwich structure. The second GNP conjugate was engineered to attach to the first GNP conjugate to achieve signal amplification. This biosensor enabled the visual and quantitative detection of exosomes through the buildup of GNP on the test zone. It demonstrated a low LOD of 1.3 × 10^3^ particles/μL, enhanced 13-fold over the standard lateral flow assay (Figure 12) [80].

**Figure 13:**
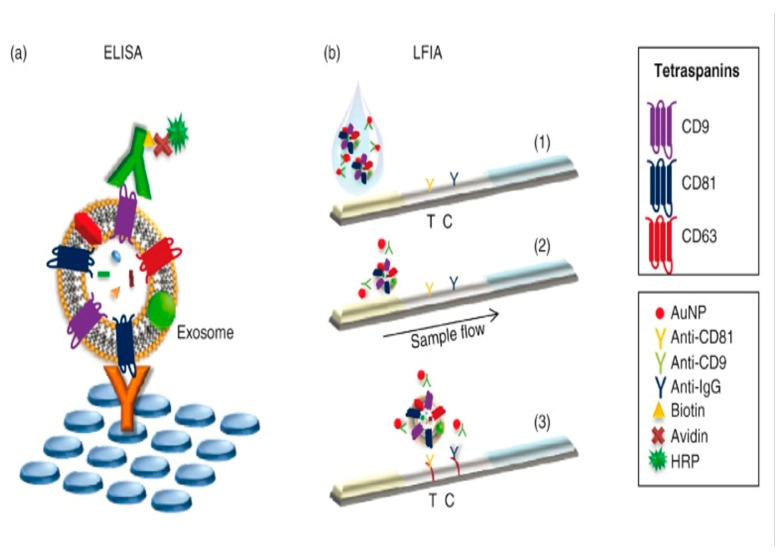
Schematic view of the experimental procedure for exosome detection. (**a**) Schematic representation of the ELISA set up for exosome detection. Here, each reagent is added in sequential steps. (**b**) Schematic representation of the lateral flow immunoassay dipstick. (**1**) Specific antibodies against tetraspanins (test, T) and anti-mouse immunoglobulin antibodies (control, C) are immobilized on the membrane. (**2**) Exosomes, if present in the sample, are detected by the detection probes (AuNP-conjugated antibodies). (**3**) As the complexes flow, they are captured onto the membrane by the immobilized antibodies [81].

Oliveira-Rodriguez et al. reported an in-house ELISA for detecting exosomes purified from cell culture supernatants and multiple lateral flow immunoassay (LFIA) for detecting exosomes separated from cell culture supernatants and commercial exosomes previously enriched from human plasma and urine. Oliveira-Rodriguez introduced a novel lateral flow with multiple applications. With an LOD of 8.54 × 10^5^ exosomes/mL, this test was used to identify exosomes extracted from a malignant melanoma cell line (Figure 13) [81].

### 3.5. Luminescence Biosensors

Luminescence is the process through which a material emits light without requiring or producing heat. Numerous luminescence-based aptamer sensors have been developed for exosome detection because of their high sensitivity, low cost, and ease of operation. Based on the energy source, luminescence is divided into two subtypes: electrochemiluminescence and luminescence resonance energy transfer.

The strengths of luminescence sensors are as follows. Luminescent sensors are particularly effective for identifying targets that might otherwise go undetected. The sensor can mark a target only visible when exposed to UV light. A luminescence sensor can differentiate between fluorescent and other highly reflective materials, as it emits UV light while detecting visible waves. Specific and capable of distinguishing between closely similar targets. Sensitive to modest quantities of target within a dense background matrix. High affinity and the ability to sustain binding even after multiple washing procedures. It is stable enough to be used for an extended period.

The lateral luminescence sensors also have some possibility of minor limitations. A significant limitation of the luminescence sensor approach with most sensor supports is the lack of selectivity when investigating multicomponent samples, as comparable chemicals may exhibit identical luminescence characteristics.

#### 3.5.1. Electrochemiluminescence Biosensors

**Figure 14:**
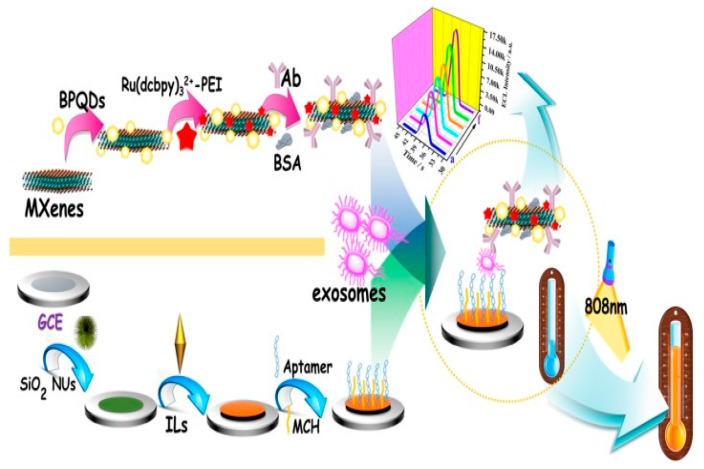
Schematic illustration of the dual-mode biosensor for exosome detection [82].

Fang et al. built an electrochemiluminescence (ECL) and photothermal dual-mode biosensor for exosome detection using black phosphorous quantum dots (BPQDs) and MXenes as signal amplifiers. Specifically, BPQDs can catalyze the oxidation of Ru(dcbpy)_3_^2+^ and be employed as a coreactant for the first time. The generated self-enhanced Ru(dcbpy)_3_^2+^@BPQDs electrochemiluminescence system can create a strong electrochemiluminescence signal by minimizing energy loss and reducing electron transfer distance. MXenes have a high specific surface area and excellent conductivity. Therefore, they were used as a supporter to increase the immobilization quantity of Ru(dcbpy)_3_^2+^ and BPQDs, which improved the electrochemiluminescence signal with a low LOD of 37 particles/μL. Moreover, BPQDs and MXenes exhibit outstanding photothermal properties and have been successfully employed as thermal conversion devices in the development of a photothermal biosensor for exosome analysis. This study used a unique dual-modality probe of MXenes-BPQDs to create a biosensor, which expanded the use of MXenes and BPQDs in bio-detection and offered an efficient and robust approach for exosome recognition with an LOD of 37 particles/μL (Figure 14) [82].

#### 3.5.2. Luminescence Resonance Energy Transfer Biosensors

**Figure 15:**
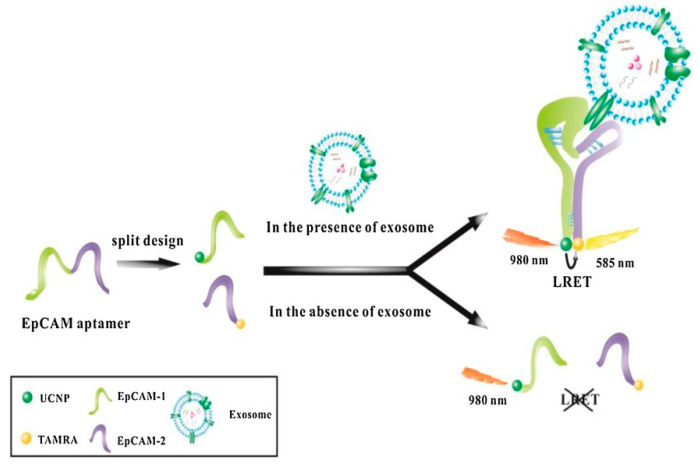
The strategy diagram of the aptasensor based on LRET between UCNPs and TAMRA [83]. A washing-free aptasensor based on LRET between UCNP donor and TAMRA acceptor for highly sensitive detection of exosomes was proposed, which could significantly simplify the testing process and shorten the detection time.

Exosome identification has promise for early cancer detection. However, developing adequate optical sensors remains difficult. Wang et al. created a washing-free aptamer sensor for extremely sensitive detection of exosomes based on luminescence resonance energy transfer (LRET) between rare-earth-doped upconversion nanoparticles (UCNPs) donor and tetramethyl rhodamine (TAMRA) acceptor. The binding of aptamers to the epithelial cell adhesion molecule (EpCAM), a highly expressed surface protein of exosomes, allows for the combination of UCNPs and TAMRA. As a result, when the UCNPs-TAMRA system was activated by near-infrared light at 980 nm in the presence of exosomes, TAMRA produced yellow fluorescence at 585 nm owing to LRET. As a result, the fluorescence intensity at 585 nm was linearly proportional to exosome concentration, making exosome detection and quantification possible. Under ideal conditions, the suggested method may achieve an LOD of 80 particles/µL while efficiently reducing the background signal by utilizing UCNPs as an energy donor (Figure 15) [83].

**Figure 16:**
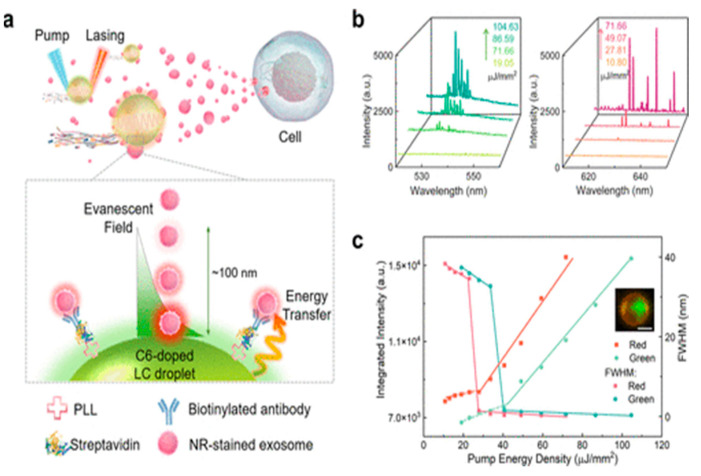
(**a**) Sensing mechanism of the self-propelled microlaser for detecting the cell-derived exosome. The inset shows the concept of interfacial energy transfer through the cavity interface from green lasing emission to red lasing emission. (**b**) Photoluminescence spectra were recorded at the donor and acceptor lasing regions after exosome binding. The left and right panels show lasing spectra under different pump fluences independently. (**c**) Full-width at half-maxima (FWHM) and derived lasing threshold plot from the green emission band (C6) and red emission band (NR). Inset: microlaser droplet after exosome binding. Scale bar: 10 μm [84].

Self-propelled micro/nanomotors, as proposed by Wang et al., are being developed as smart sensors for evaluating extracellular biomarkers in circulating biofluids. Traditional luminous motors are sometimes obscured by a highly dynamic and dispersed environment, making it difficult to identify biomarkers or delicate binding dynamics. Wang et al. presented a method for amplifying small signals by coupling strong light-matter interactions on micromotors. A liquid crystal microdroplet shows a smart whispering-gallery-mode microlaser that can self-propel and evaluate extracellular biomarkers. Exosomes produced from 3D multicellular cancer spheroids were labeled with long-lasting spectral responses. These responses were induced by cavity energy transfer, yielding unique molecular labels for cellular profiling. Lastly, a microfluidic biosystem containing several tumor-resultant exosomes. It was used to develop its sensing capabilities in challenging situations further (Figure 16) [84].

### 3.6. Colorimetric Biosensors

A colorimetric aptamer sensor allows for a readout response with direct observation of a “yes/no” answer. This can be easily conducted without specialized sensor devices, offering low-cost, rapid, and easy identification.

The strengths of colorimetric sensors include their low cost, quick response time, and ease of use. It is a more convenient procedure than volumetric or gravimetric processes and is easily adjusted for automation. It does not necessitate the use of an expert. It was used for the quantitative analyses of colored substances. Colorimetry also has the benefit of being a portable system that is easy to carry and transfer.

The colorimetric sensors also have some possibility of minor limitations. The main drawback of colorimetric sensing is that it cannot evaluate colorless molecules. More sample is required for analysis. A standard solution must be prepared. It has a modest sensitivity. The same colors from conflicting material may cause findings to be incorrect. For more accurate analysis, the specific wavelength bandwidth may be necessary. In uncontrolled situations, tampering with the matrix might lead to depraved outcomes.

**Figure 17:**
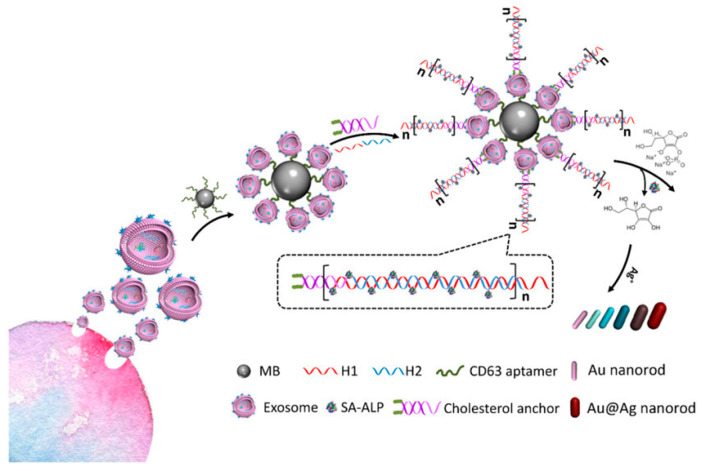
Schematic illustration of the mechanism for multicolor visual detection of exosomes based on HCR and enzyme-catalyzed metallization of Au NRs [85].

Exosome detection using a sensitive multicolor visual technique built on enzyme-induced silver deposition on gold nanorods was developed by Zhang et al. [85]. Hybridization chain reaction (HCR) incorporated additional alkaline phosphatase (ALP) for signal amplification to obtain extremely sensitive exosome determination. After capturing exosomes with an MB-labeled CD63 aptamer, cholesterol-altered DNA probes were instinctively incorporated into the exosomal lipid membrane. The DNA probe ends function as the signal amplification initiator by triggering the HCR. Finally, increasing the number of sites resulted in higher ALP loading, and ascorbic acid synthesis was increased, facilitated by HCR. Ascorbic acid decreased silver ions, and silver shells developed on gold nanorods, resulting in the blue shift of the longitudinal localized SPR peak. Similarly, the concentration of exosomes may be identified with the naked eye due to the dramatic color change. Because of the combined signal amplification of HCR and the metallization of Au NRs, very sensitive detection of exosomes was achieved, with detection limits as low as 1.6 × 10^2^ particles/µL by UV–visible spectroscopy and 9 × 10^3^ particles/µL by naked eyes (Figure 17).

**Figure 18:**
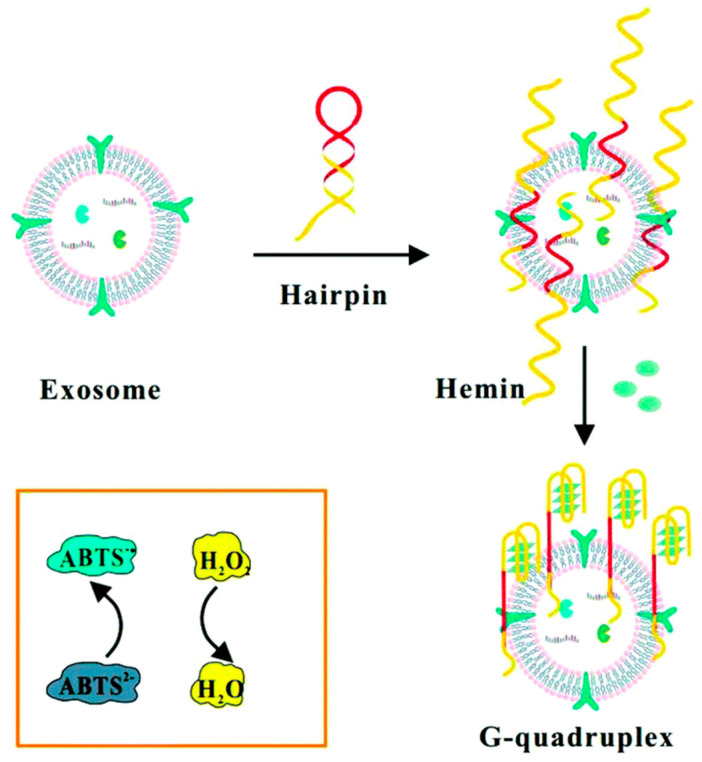
Schematic illustration of the assay for the detection of exosomes by binding to hairpin structures based on the combination of the MUC1 aptamer and G-quadruplex-mimetic enzyme [86].

Zhou et al. designed a hairpin-like structure that linked the highly specific Mucin 1 aptamer with a hemin/G-quadruplex to detect breast cancer exosomes. It resulted in a sensitive, simple, and low-cost colorimetric aptamer sensor. Because it acts as a Horseradish peroxidase-mimicking DNAzyme, the hemin/G-quadruplex toward H_2_O_2_ reduction produced a significant colorimetric reaction. The aptamer sensor was thought to be an “on-off” switch that rigorously controls the reaction process in response to exosomes’ presence or absence [86]. This breast cancer exosome detection method detected an LOD of 3.94 × 10^5^ particles/mL (Figure 18).

**Figure 19:**
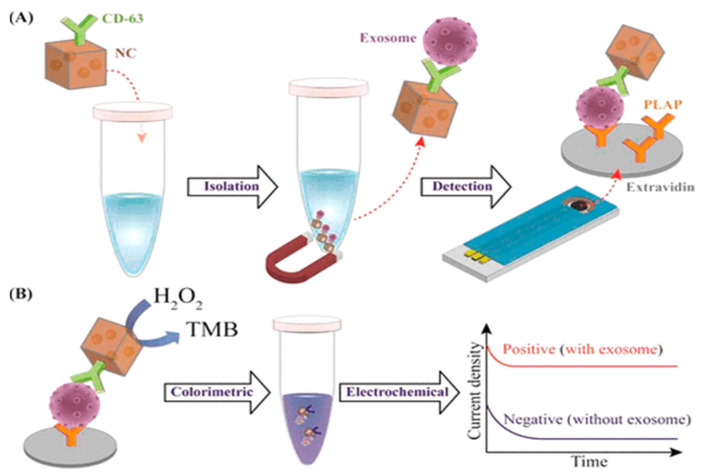
Schematic representation of the assay for direct exosome isolation and detection from cell culture media. In this method, the Au-NPFe_2_O_3_NC were initially functionalized with a generic antibody (CD63) and dispersed in sample fluids (cell culture media) to capture bulk exosomes. After magnetic capture and purification, exosome-bound Au-NPFe_2_O_3_NC have transferred to PLAP antibody-modified, screen-printed electrodes. The peroxidase-like activity of Au-NPFe_2_O_3_NC was then used to achieve naked-eye detection along with UV–visible and electrochemical quantification of PLAP-specific exosomes present in the cell culture media [87].

Boriachek et al. [87] provide a simple method for direct separation and subsequent detection of a particular population of exosomes utilizing gold-loaded ferric oxide nanocubes (Au-NPFe_2_O_3_NC), a manufactured superparamagnetic material with multifunctional capabilities. In this procedure, the Au-NPFe_2_O_3_NC was first functionalized with a generic tetraspanin (exosomes-associated) antibody (i.e., CD63) before being disseminated in sample fluids and acting as “dispersible nanocarriers” to capture the majority population of exosomes. After magnetic collection and purification, exosomes attached to Au-NPFe_2_O_3_NC were transported to the tissue-specific, antibody-modified, screen-printed electrode. Boriachek et al. employed a unique placental marker, placenta alkaline phosphatase (PLAP), to identify exosomes released by placental cells as a proof of principle. The peroxidase-like activity of Au-NPFe_2_O_3_NC was subsequently used to develop an ELISA-based sensing methodology for naked-eye inspection and UV–visible and electrochemical detection of PLAP-specific exosomes in the placental cell-conditioned medium. Boriachek et al. found excellent agreement in analytical performance for the detection of placental cell-derived exosomes (i.e., linear dynamic range, 10^3^–10^7^ exosomes/mL; LOD, 10^3^ exosomes/mL; relative standard deviation (%RSD) of <5.5% for (*n* = 3) when using a commercial “total exosome isolation kit”-based pre-isolation step (Figure 19).

**Figure 20:**
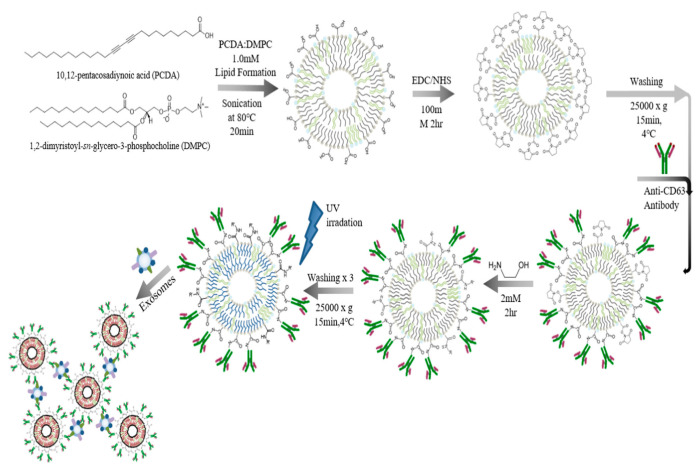
Schematic illustration of the fabrication of the PDA liposome immunosensor for exosome detection. The liposome consisted of a PCDA monomer and a DMPC phospholipid. PDA liposomes, composed of PCDA and phospholipid, were activated by EDC/NHS chemistry, and antibodies were attached to the PDA liposome, conferring selectivity to the PDA liposome for exosome detection. After adding exosomes, the PDA vesicle solution undergoes color change and fluorescence [88].

Kim et al. created a liposomal biosensor based on polydiacetylene (PDA), a conjugate polymer with distinctive optical characteristics widely employed in sensing applications. To improve selectivity and sensitivity to sensory input, antibodies targeting CD63, a membrane protein present only in exosomes, were linked to PDA liposomes, and phospholipid molecules were inserted into PDA vesicles. By detecting colorimetric changes caused by the ligand–receptor interaction of PDA vesicles, the signal analysis produced from PDA liposomes for exosome identification and quantification was carried out. Signals from the PDA lipid immunosensor were obtained using visual, UV–visible, and fluorescence spectroscopic techniques, yielding a 3 × 10^8^ vesicles/mL LOD. This minimal concentration was employed in practical applications (Figure 20) [88].

### 3.7. Electrochemical Biosensors

The aptamer is used as the biological recognition element and is combined into the surface of an electrochemical sensor. The sensor produces assessable electrical signals when it reacts with the target material of interest (biological recognition element). This aptamer sensor boasts remarkable features, including high-sensitivity detection, affordable, and easy-to-use instrumentation with automatic setup, and excellent mobility for clinical settings.

The strengths of electrochemical sensors include their ability to be tailored particularly for a given analyte and their range in the sample space. The analyte concentration determines the degree of selectivity. Straight performance, low power consumption, linear output, and high resolution. Additional advantages include remarkable repeatability and precision. Once calibrated to a known analyte concentration, the sensor will accurately read a repeated target analyte. Other cross-interferences do not affect the output of these sensors. The presence of other analytes in the environment does not affect the sensor’s life. Most alternative analyte detection methods, such as infrared and photoionization detectors, are less costly. Electrochemical sensors are both practical and cost effective.

The electrochemical sensors also have some possibility of minor limitations. Temperature range is limited or constrained. Temperature is a crucial element for electrochemical sensors, which are typically internally temperature compensated. It is best to maintain a constant temperature as possible. Short or restricted shelf life is one of the flaws. An electrochemical sensor normally has a shelf life of six months to one year, depending on the application and environmental conditions. The cross-affectability of distinct analytes is also a flaw. Though this is a benefit, it is also a drawback. If two analytes are physically similar, certain sensors may interfere with each other. Understanding which analytes interact with the sensor is critical to avoid misleading results. The life span is reduced due to increased exposure to the target analyte. Electrochemical sensors typically have a life expectancy of one year. Low humidity, high temperatures, exposure to target analytes, and cross-sensitivity to other analytes may cause the sensors’ electrolytes to dry up and deplete.

**Figure 21:**
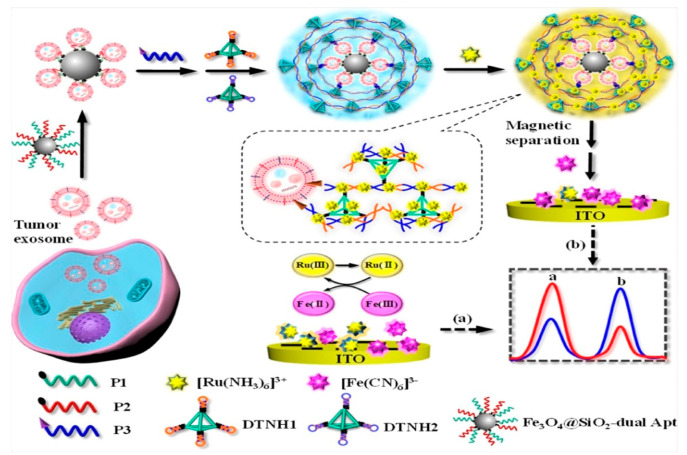
Schematic illustration of the ratiometric immobilization-free electrochemical sensing system for tumor exosome detection (**a**) in the absence and (**b**) in the presence of the tumor exosomes [89].

Yang et al. designed a ratiometric, immobilization-free electrochemical sensing device capable of accurately capturing and immediately quantifying tumor exosomes in a complex biological environment. The analytical results showed that the capture efficiency of the produced Fe_3_O_4_@SiO_2_-dual Apt NPs was better than that of single-aptamer-functionalized Fe_3_O_4_@SiO_2_ NPs. As a result, the capacity of a dual-aptamer identification system with strong capture ability has been validated to detect and separate tumor exosomes effectively. Very sensitive and selective detection of tumor exosomes was achieved using this dual-aptamer identification method and hyperbranched DNA superstructure signal amplification technique, with an LOD of 3.0 × 10^4^ particles mL^−1^ (Figure 21) [89].

**Figure 22:**
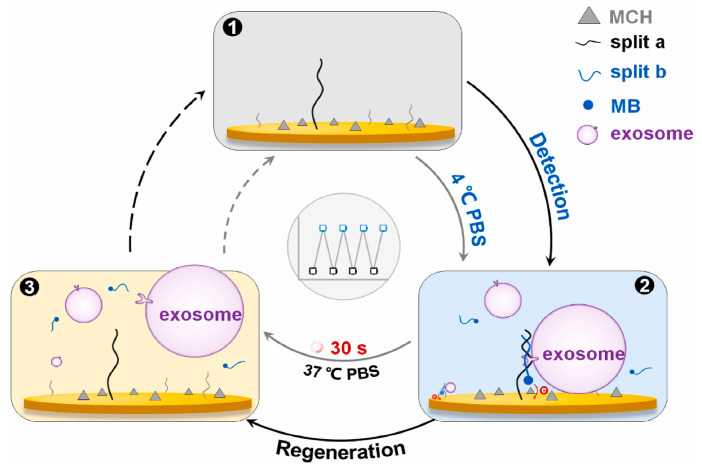
Schematic illustration of split-aptamer mediated regenerable temperature-sensitive (SMRT) electrochemical biosensor for the detection of tumor exosomes [90].

Liu et al. developed a split-aptamer-mediated regenerable temperature-sensitive (SMRT) electrochemical biosensor for exosome detection. The split-aptamer employed in this SMRT biosensor comprised two fragments, one immobilized on the surface of an electrode through sulfhydryl groups and was dubbed “split-a,” while the other was dubbed “split-b.” The two fragments created sandwich structures at the electrode surface by target-induced self-assembly in the presence of target exosomes in PBS at 4 °C and subsequently detected exosomes using voltammetry. Furthermore, due to the split-temperature aptamer’s sensitivity, the electrode might be renewed by the temperature-induced breakdown of the sandwich structures. As a result, the SMRT biosensor performed a sensitive and specific analysis of target exosomes with an LOD of 1.5 × 10^6^ particles/mL. It could be swiftly regenerated by washing with PBS at 37 °C for 30 s without any additives (Figure 22) [90].

**Figure 23:**
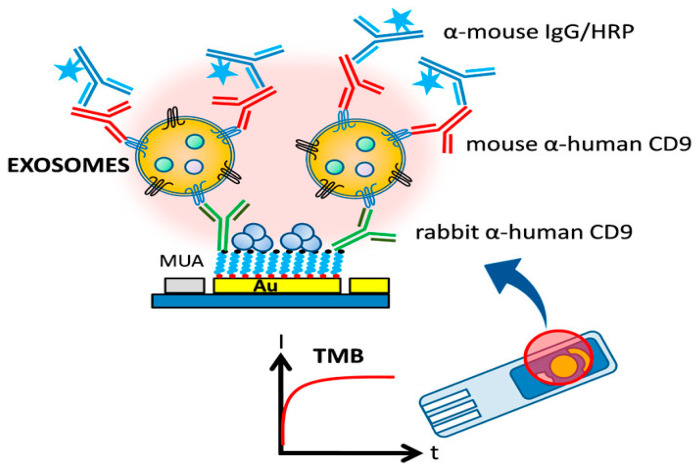
Electrochemical sandwich immunosensor for determination of exosomes based on surface marker-mediated signal amplification [91].

Doldán et al. propose an exosome detection technique utilizing gold electrodes functionalized with α-CD9 antibodies. Once the sample was placed on the electrodes, the collected exosomes were examined using a separate organism’s α-CD9 antibody. This technique enables effective signal amplification, as multiple copies of the CD9 protein are exposed on the surface of each exosome. Finally, a horseradish peroxidase (HRP)-conjugated-mouse IgG antibody was used, and the electrochemical reduction of HRP-oxidized TMB was seen. With an LOD of 200 particles/µL, the sandwich-type electrochemical immunoassay distinguishes between exosomes and other EVs (i.e., microvesicles). It has a dynamic range spanning over 4 orders of magnitude (Figure 23) [91].

**Figure 24:**
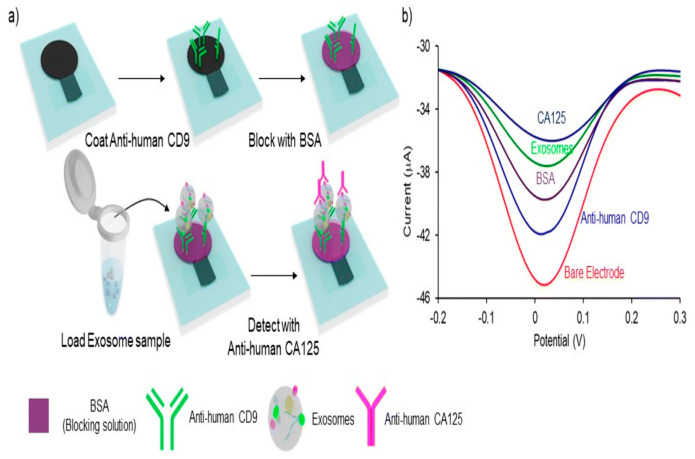
Schematic diagram of the sandwich immunological assay for cancer cell-derived exosome detection. (**a**) The exosomes were extracted from the cell culture media and spiked in PBS buffer. CD9 antibodies were immobilized on PCEs by protein adsorption on the paper matrix. Total exosome populations were captured via their immune interaction with surface-bound CD9 antibodies. Ovarian cancer cell-derived exosomes were sub-populated by using CA-125 antibodies. (**b**) DPV signal depicting the stepwise attachment of each layer on the PCEs surface [92].

Kasetsirikul proposed a cost-effective electrochemical paper-based analytical instrument for the measure. Using a study finding, the instrument detects total availability and cancer cell-derived exosomes inside the cell culture medium. The instrument incorporates a sandwich immune assay design. The instrument first caught exosomes using electrode-bound generic antibodies (e.g., CD9) and then identified them using ovarian cancer-specific CA125 antibodies. Through a relative standard deviation of 10, the proposed device from Kasetsirikul measures overall total exosome concentration with an LOD of 9.3 × 10^7^ exosomes/mL. The ovarian cancer cell-derived exosomes LOD of 7.1 × 10^8^ exosomes/mL was detected (Figure 24) [92].

### 3.8. Fluorescent Biosensors

Fluorescence biosensors are molecules and systems that use fluorescence to assess the concentrations, positions, and other dynamics of biomolecules and bioactivities.

The strengths of fluorescent sensors include portability, which enables their widespread utilization in various applications, including environmental monitoring, disease diagnostics, drug development, and food quality monitoring. Fluorescence-based portable biochemical sensors are acknowledged as having significant advantages over other technologies due to their high sensitivity, selectivity, fast response, and ease of use. Unlike colorimetric or absorbance-based sensing approaches, the fluorescence signal is detected immediately without reference beam comparison. As a result, fluorescence-based detection is frequently a more appealing alternative for field applications when only a limited number of biochemical samples are available.

The fluorescent sensors also have some possibility of minor limitations. Despite the wide variety of probes available, fluorescence-based sensors are constrained by their reliance on probes. Unlabeled and incapable of autofluorescence, molecular structures will stay black in the final picture. So, unexpected or novel structures cannot be noticed without prior identification and proper probe design or selection. Furthermore, the use of many probes at the same time, or the presence of autofluorescent biomolecules, may cause interactions that reduce the efficiency of each probe, resulting in a worse signal-to-noise ratio. Many chemicals occurring naturally within the target and other fluorophores can quench a fluorescence signal.

**Figure 25:**
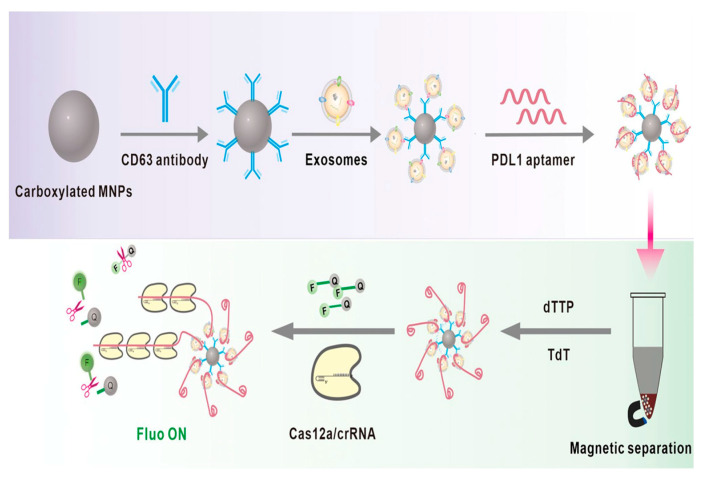
Schematic of a fluorescence biosensor for detection of exosomes [93].

For the detection of nasopharyngeal cancer (NPC)-derived exosomes, a fluorescent aptamer sensor based on the combination of magnetic nanoparticles (MNPs), terminal deoxynucleotidyl transferase (TdT), and CRISPR/Cas12a was developed by Yi et al. Due to their magnetic separation capacity, MNPs can reduce background interference. TdT can create an ultra-long polynucleotide tail that binds to several crRNAs, amplifying the signal. CRISPR/Cas12a trans-cleavage activity may be precisely induced by crRNA binding to DNA, leading to the cleavage of a bi-labeled DNA reporter containing fluorescence and quencher. The fluorescence spectra’s excitation wavelength was 490 nm. Fluorescence spectra were acquired with emission wavelengths spanning from 511 to 600 nm. The built fluorescent aptamer sensor for NPC-derived exosome identification demonstrated high sensitivity and specificity under optimization conditions, with a linear range of 500 to 5 × 10^4^ particles mL^−1^ and an LOD of 100 particles mL^−1^ (Figure 25) [93].

**Figure 26:**
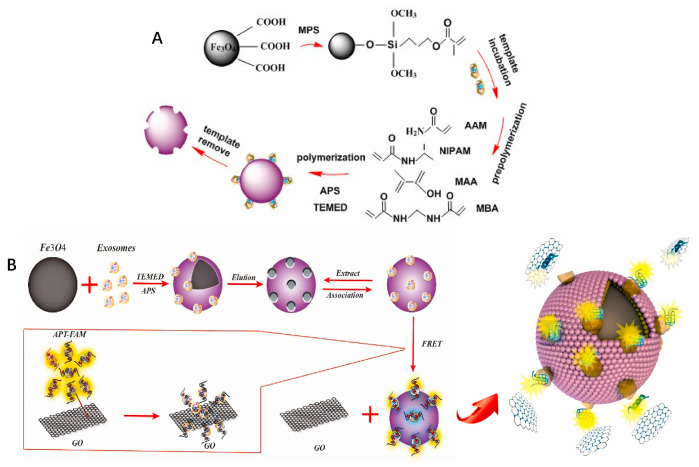
A dual-selective sensor for exosomes in serum using magnetic imprinted polymer isolation sandwiched with aptamer-/graphene oxide-based FRET fluorescent ignition. (**A**) Demonstration of the chemical reactions for MIP preparation; (**B**) the schematic illustration of details of the experimental principle [94].

Feng et al. created a novel molecularly imprinted “turn-on” fluorescent biosensor. They combined MIP and an aptamer/graphene oxide (GO)-based fluorescence resonance energy transfer (FRET) system for optical sensing in a sandwich mode. The sensor fluorescence intensity of Lyz in blood changed linearly with the concentration of Lyz. It ranged from 1.19 × 10^−6^ to 4.76 × 10^−5^ mol/L (R^2^ was 0.9816). It also showed an LOD of 2.27 μmol/L. The recovery rate was 104.17%. The fluorescence intensity of the device displayed a unique linear relationship with the exosome concentration. The LOD of 2.43 × 10^6^ particles/mL, when the total protein of the exosomes was injected into exosome-lacking plasma, was in the range of 0.88–8.80 mg/mL (Figure 26) [94].

**Figure 27:**
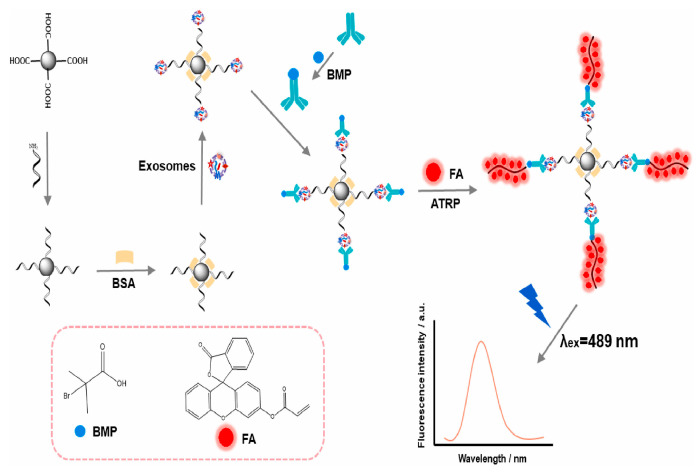
Schematic diagram of highly sensitive fluorescent sensor detection of A549 exosomes based on ARGET ATRP signal amplification strategy [95].

Yang et al. demonstrated a sensitive method for enhancing exosome fluorescence measurement utilizing an activator regenerated by electron transfer (ARGET) to atom transfer radical polymerization (ATRP) polymerization. CD63 aptamer and EGFR antibody specifically detected A549 cell-derived exosomes, providing good specificity and accuracy to the proposed sensor. Furthermore, the fluorescence signal strength was increased by introducing a high number of fluorescent molecules of fluorescent antibody through an activator regenerated by electron transfer ATRP (ARGET ATRP) polymerization. The sensor performed well analytically, with a linear range of 5 × 10^4^ exosomes/mL to 5 × 10^9^ exosomes/mL and an LOD as low as 11,610 exosomes/mL. Additionally, this fluorescent technique has good selectivity and anti-interference capability (Figure 27) [95].

**Figure 28:**
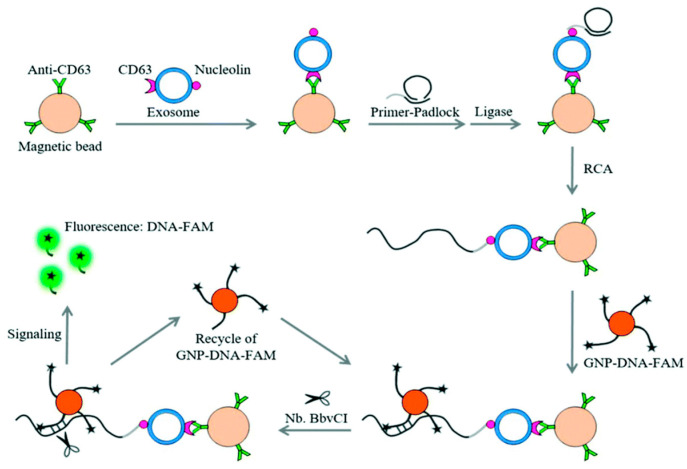
Schematic illustration of the dual-signal amplification-based platform for the ultrasensitive detection of exosomes. The primer contains the RCA primer (labeled in black) and AS1411 aptamer (labeled in gray) [96].

Huang et al. developed a fluorescent biosensing device that utilizes dual signal amplification to detect leukemia cell-derived exosomes with high sensitivity. The procedure was divided into the following steps: First, leukemia-derived exosomes containing CD63 and nucleolin were captured using anti-CD63 antibody-modified MB conjugates (MB-CD63); next, a DNA primer containing a nucleolin-recognition aptamer (AS1411) was used to bind the exosomes, triggering a rolling circle amplification (RCA) reaction to generate many repeat sequences for hybridization with gold nanoparticle (GNP)-DNA-fluorescent dye (FAM) conjugates (GNP–DNA–FAM). Consequently, FAM was liberated from GNP-DNA-FAM conjugates, the quenching state was converted to the emission state, and fluorescence signals were continually accumulated. The LOD of 1 × 10^2^ particles/L exosomes LOD was identified using this signal amplification technology (Figure 28) [96].

## 4. The Comparison Between Advantages and Disadvantages of Antibody-Based Sensors and Aptamer-Based Sensors

Antibody-based sensors have long been the foundation of biomolecular recognition in biosensing technologies for exosome detection. Their main benefit is their extraordinary specificity and affinity for target antigens, which result from a naturally formed immune recognition mechanism. The intricate three-dimensional structure of antibodies, which includes multiple binding regions (Fab fragments) and complementarity-determining regions, allows for precise targeting of exosomal surface proteins such as CD9, CD63, and disease-specific markers. This robust specificity enables antibody-based sensors to give high analytical accuracy in complicated biological samples, allowing for accurate detection even at low exosome concentrations. Furthermore, antibodies have a long history in a variety of sensor formats, including electrochemical, SPR, colorimetric, and lateral flow strip platforms, which improves their dependability, commercial availability, and acceptance in clinical practice. However, antibody-based sensors have significant shortcomings. High-quality antibody manufacturing is biologically intense, time consuming, and expensive, frequently involving animal immunization and hybridoma technology. Antibodies can vary from batch to batch and are susceptible to denaturation or degradation, particularly under harsh conditions such as high pH, temperature variations, or exposure to organic solvents—all of which can have a negative impact on sensor stability and shelf life. Furthermore, their relatively large molecule size may limit surface coverage density on sensor platforms, compromising sensitivity and quick kinetics when compared to synthetic alternatives. Despite their specificity, antibodies’ cross-reactivity with structurally identical non-target molecules can diminish selectivity, and their reusability in sensors is frequently limited due to irreversible binding or sensor fouling.

Aptamer-based sensors, on the other hand, offer a promising alternative by employing synthetic single-stranded DNA or RNA sequences that fold into distinct three-dimensional structures capable of binding exosomal proteins and markers with great affinity and selectivity. Aptamers provide several significant advantages over antibodies. They are created using in vitro SELEX (Systematic Evolution of Ligands by Exponential Enrichment), which is faster, cheaper, more reproducible, and free of animal-derived ethical concerns. Aptamers can be adjusted for stability, size, and modifiability through chemical synthesis, resulting in increased physical and chemical resilience: they stay functional throughout a wide pH and temperature range and can be regenerated for various applications using simple denaturation-renaturation methods. Their tiny size enables larger sensor surface densities, which improves sensitivity and signal transduction. Aptamers can also be subjected to a broader range of chemical changes, enabling for integration with innovative nanomaterials, smart delivery systems, and multiplexed sensor designs, as illustrated by the numerous magnetic, SPR, SERS, colorimetric, and fluorescent approaches examined. Nevertheless, aptamer-based sensors have drawbacks. The selection of truly high-specificity aptamers for specific targets can be difficult, and aptamers are vulnerable to nuclease degradation in biological fluids unless chemically modified for protection. Aptamer binding affinities may fall short of the best monoclonal antibodies in some circumstances, lowering detection limits in highly complicated or low-abundance materials. Competing nucleic acids, secondary structures, and buffer composition changes can all have an impact on aptamer performance. Finally, while aptamer technology is quickly advancing, it does not yet have the clinical validation and regulatory track record that antibody-based approaches possess. As a result, while deciding between antibody- and aptamer-based exosome sensors, consider the intended application, required operational environment, cost restrictions, and the necessity for sensor reusability, multiplexing, and stability. The comparison of aptamer- and antibody-based sensors for exosome detection is shown in Table 2.

## 5. Presently Demonstrated and Upcoming Applications

Antibodies and aptamers are employed in biosensor functionalization for disease detection, which directly contributes to early health retrieval and patient survival. It is also useful for planning disease prevention efforts. More effective illness detection systems may help reduce death rates by providing a better understanding of disease progression and enabling early access to individualized medical therapy. EVs identification and detection biochip offers a reliable and rapid liquid biopsy platform for the analysis of complex biofluids. It offers EVs isolation and detection in a single chip using a small sample volume of 300 µL and an assay time of 1.5 h [97]. Several approaches to early disease detection that use aptamers or antibodies as ligands have been presented in the literature. Most are still in the lab testing phase, but preliminary results suggest they could be successful outside of the lab.

Along with a single aptamer sensing technology, dual-mode aptasensors are also getting developed, and the technology represents a significant advancement in exosome biosensing technology by integrating two complementary detection modalities within a single analytical platform. This synergistic architecture leverages the high specificity of aptamers for exosome surface proteins with orthogonal signal transduction mechanisms. It commonly combines electrochemical and optical, or fluorescence and colorimetric outputs to markedly enhance sensitivity, robustness, and clinical reliability [82,83,89]. The dual-mode strategy addresses key challenges inherent to exosome analysis in complex biological matrices; by enabling simultaneous or sequential measurement of independent physical properties, it dramatically reduces susceptibility to matrix effects, cross-reactivity, and false positives, while broadening the dynamic detection range. Typically, exosome binding by a specific aptamer induces conformational changes or fosters sandwich-like assemblies, triggering distinct and complementary signal outputs for each detection channel. For instance, Fang et al. engineered a dual-mode sensor integrating ECL and photothermal readouts, enabled by black phosphorus quantum dots and MXene nanosheets as dual-function signal amplifiers, achieving exosome LOD as low as 37 particles/μL and offering robust signal confirmation [82]. In a related approach, Wang et al. exploited both LRET and conventional fluorescence using aptamer-modified upconversion nanoparticles; this facilitated a wash-free, quantitative method with an LOD of 80 particles/μL and outstanding background suppression [83]. Similarly, Yang et al. demonstrated a dual-aptamer, immobilization-free, ratiometric electrochemical sensor for direct exosome quantification, overcoming many sample matrix interferences and achieving ultrasensitive and reproducible responses [89]. Collectively, these innovations underscore the transformative potential of dual-mode aptasensors, not only for their heightened analytical performance and cross-validated outputs but also for their adaptability to point-of-care and microfluidic formats, paving the way for robust, early-stage, and non-invasive exosome-based diagnostics.

This review addresses a cutting-edge data-gathering gap and examines the scope of enhancements for future research. This review paper discusses research on aptamer and antibody biosensors for detecting disease-specific exosomes in diverse body fluids. This study also described advancements in single-stranded aptamer-based and antibody-based biosensors for detecting various disease-derived exosomes. Aptamers and antibodies have distinct features that make them ideal for aptamer and antibody sensor development, biological identification, and signal transduction. These techniques have significantly enhanced the sensitivity of exosome detection by integrating it with adaptive technologies, such as magnetic, SPR, SERS, lateral flow strip, luminescence, colorimetric, electrochemical, or fluorescent methods, on functionalized biosensors. Furthermore, detection equipment is becoming increasingly compact and user friendly, featuring new portable sensors, smartphone-based scanners, and corresponding Android or iOS apps. The growing literature in the biosensing field suggests that using aptamer and antibody sensors could be a viable alternative for improving the efficiency of sickness detection and monitoring.

Furthermore, there is a growing interest in studying changes in the simple structural structure of exosomes and their relationship to disease detection. New exosomal biomarkers must also be discovered and validated for the specific identification of diseases. Increased collaboration among worldwide diagnostics research, including aptamer-/antibody-based biosensors, is expected to result in further advancements through transdisciplinary interactions. Aside from detecting exosomes, aptamers, and antibodies have gained attention for their prospective application as an exosomal delivery mechanism for theranostic-targeted drug administration.

## 6. Future Trends

Despite the employment of aptamers and antibodies in various sensing platforms, significant technological challenges and limitations must be addressed to enhance detection capabilities. Given the complex background of real-world samples, a technique for point-of-care diagnostics is necessary, particularly in mitigating the interference of all non-targeted biomarkers from surrounding live cells. Meanwhile, the advancement of exosome isolation, enrichment, purity, and easy-release technology requires major effort for clinical diagnostic applications and physiological research. It comprises recently developed aptamer or antibody-based size-selective separation technologies.

The quantity and quality of ongoing research on exosome detection with aptamers and antibodies are excellent. Nonetheless, researchers must extensively examine the sensitivity, lower and higher limits of detection, and lower limits of detection to boost detection sensitivity, reduce per-test costs, ensure test results are repeatable, and make biosensors reusable. These biosensing technologies should be tested in clinical settings using real clinical human samples from large disease-specific patient populations with various genetic origins. The majority of published research has focused on biosensing in buffered or diluted biological fluid samples. The objective should be to detect biomarkers in biologically active human samples. The impact of non-targeted exosomal biomarkers from surrounding living cells on the selectivity of the designed aptamer/antibody biosensors should be investigated.

## 7. Conclusions

The research should discuss the changes in biosensor behavior in non-laboratory and household environments. The user friendliness, biosensor-related precautions, and discard protocol should all be listed. While including multiple biosensing components may result in higher detection limits, the increased complexity of the multicomponent system may impede clinical translation. Paper-based or degradable material-based lateral flow strip biosensors, combined with simple mobile app readers, may be suitable for doctor-/hospital-based clinical testing. The cost of biosensor equipment, including storage and calibration, must also be considered. To avoid clinical accidents, the use of components such as lasers or X-rays is usually restricted. Concentrating on these practical challenges may speed up the transition of these promising platforms from research labs to clinics. We are confident that the information obtained from biosensing platforms has the potential to provide reliable healthcare information on an individual patient’s prognosis and will assist individuals in achieving personalized prophylactic goals.

## Figures and Tables

**Table 2:** Comparison of aptamer- and antibody-based sensors for exosome detection.

Features	Aptamer-Based Sensor	Antibody-Based Sensor
Recognition Element	Aptamer (ssDNA or RNA)	Antibody
Production	Chemical synthesis, fast, cost effective	Biological (animals/cells), slow, costly
Specificity/Affinity	High, can be tuned	High, well characterized
Stability	More stable, resists harsh conditions	Sensitive to heat, pH, organic solvents
Modifiability	Flexible chemical modification	Limited modification options
Batch Variability	High reproducibility	Can vary between batches
Shelf Life	Long (especially if modified)	Short to moderate
Size	Small (≈8–25 kDa)	Large (≈150 kDa)
Reusability	High (can be regenerated)	Limited (may denature)
Nuclease Sensitivity	Sensitive (but can be chemically protected)	Not applicable
Clinical Acceptance	Gaining acceptance	Well-accepted, validated
Cost per assay	Low to moderate	Moderate to high
Common Sensor Types	All (SPR, SERS, LFA, etc.)	All (SPR, SERS, LFA, etc.)
Limitations	Nuclease sensitivity, sometimes lower affinity	Costly, stability issues

## Data Availability

Not applicable.

## Abbreviations

The following abbreviations are used in this manuscript:

EV	Extracellular vesicle
MVB	Multivesicular body
ILVs	Intraluminal vesicles
Lyz	Lysosomes
SPR	Surface Plasmon Resonance
SERS	Surface-Enhanced Raman Scattering
CSF	Cerebrospinal fluid
SEC	Size-exclusion chromatography
FFF	Field-flow fractionation
TFF	Tangential flow filtration
SELEX	Systematic Evolution of Ligands by EXponential Enrichment
H	Heavy
L	Light
V	Variable
C	Constant
MB	Magnetic beads
iMAGE	Integrated Magnetic Analysis of Glycans in Extracellular Vesicles
LOD	Limit of detection
AuNP	Gold nanoparticle
Au@PDA NP	Polydopamine-functionalized gold nanoparticle
DTPs	DNA tetrahedron probes
RIU	Refractive index unit
EGFR	Exosomal epidermal growth factor receptor
PD-L1	Programmed death-ligand 1
NSCLC	Non-small cell lung cancer
iNPS	Intravesicular nanoplasmonic system
HER2	Human epidermal growth factor receptor 2
GC-SPR	Grating-coupled surface plasmon resonance
GSSNTs	Gold–silver–silver core-shell shell nanotrepangs
GNT	Gold surface nanotrepangs
AuNS@4-MBA@Au	Gold nanostar@4-mercaptobenzoic acid@nanoshell structures
B-Chol	Bivalent cholesterol
H-SERS	Hierarchical-SERS
MEDP	Magnetic beads @ exosomes @ SERS detection probe
LRG1-Exosomes	LRG1-positive exosomes
GPC1-Exosomes	GPC1-positive exosomes
AUC	Area under the operating characteristic curve
GNPs	Gold nanoparticles
D-LFA	Double gold nanoparticles (GNPs) conjugates based lateral flow assay
LFIA	Lateral flow immunoassay
ECL	Electrochemiluminescence
BPQDs	Black phosphorous quantum dots
Ru(dcbpy)_3_^2+^	Tris (4,4′-dicarboxylicacid-2,2′-bipyridyl) ruthenium(II) dichloride
BPQDs	Black phosphorus quantum dots
Ru(dcbpy)_3_^2+^@BPQDs	Tris (4,4′-dicarboxylicacid-2,2′-bipyridyl) ruthenium(II) dichloride @ Black phosphorus quantum dots
LRET	Luminescence resonance energy transfer
UCNPs	Upconversion nanoparticles
TAMRA	Tetramethyl rhodamine
EpCAM	Epithelial cell adhesion molecule
HCR	Hybridization chain reaction
ALP	Alkaline phosphatase
Au-NPFe_2_O_3_NC	Gold-loaded ferric oxide nanocubes
PLAP	Placenta alkaline phosphatase
PDA	Polydiacetylene
SMRT	Split-aptamer mediated regenerable temperature-sensitive
HRP	Horseradish peroxidase
NPC	Nasopharyngeal cancer
MNPs	Magnetic nanoparticles
TdT	Terminal deoxynucleotidyl transferase
GO	Graphene oxide
FRET	Fluorescence resonance energy transfer
ARGET	Activator regenerated by electron transfer
ATRP	Atom transfer radical polymerization
MB-CD63	Anti-CD63 antibody-modified MB conjugates
RCA	Rolling circle amplification
GNP–DNA–FAM	Gold nanoparticle (GNP)-DNA-fluorescent dye (FAM) conjugates
